# Integrated single-cell and bulk RNA sequencing revealed the molecular characteristics and prognostic roles of neutrophils in pancreatic cancer

**DOI:** 10.18632/aging.205044

**Published:** 2023-09-19

**Authors:** Biao Zhang, Jiaao Sun, Hewen Guan, Hui Guo, Bingqian Huang, Xu Chen, Feng Chen, Qihang Yuan

**Affiliations:** 1 Department of General Surgery, The First Affiliated Hospital of Dalian Medical University, Dalian, Liaoning, China; 2 Department of Urology, The First Affiliated Hospital of Dalian Medical University, Dalian, Liaoning, China; 3 Department of Dermatology, The First Affiliated Hospital of Dalian Medical University, Dalian, Liaoning, China; 4 Laboratory of Integrative Medicine, The First Affiliated Hospital of Dalian Medical University, Dalian, Liaoning, China; 5 Institute (College) of Integrative Medicine, Dalian Medical University, Dalian, Liaoning, China

**Keywords:** pancreatic cancer, neutrophils, tumor microenvironment, single-cell RNA sequencing, prognostic model

## Abstract

Pancreatic cancer, one of the most prevalent tumors of the digestive system, has a dismal prognosis. Cancer of the pancreas is distinguished by an inflammatory tumor microenvironment rich in fibroblasts and different immune cells. Neutrophils are important immune cells that infiltrate the microenvironment of pancreatic cancer tumors. The purpose of this work was to examine the complex mechanism by which neutrophils influence the carcinogenesis and development of pancreatic cancer and to construct a survival prediction model based on neutrophil marker genes. We incorporated the GSE111672 dataset, comprising RNA expression data from 27,000 cells obtained from 3 patients with PC, and conducted single-cell data analysis. Thorough investigation of pancreatic cancer single-cell RNA sequencing data found 350 neutrophil marker genes. Using The Cancer Genome Atlas (TCGA), GSE28735, GSE62452, GSE57495, and GSE85916 datasets to gather pancreatic cancer tissue transcriptome data, and consistent clustering was used to identify two categories for analyzing the influence of neutrophils on pancreatic cancer. Using the Random Forest algorithm and Cox regression analysis, a survival prediction model for pancreatic cancer was developed, the model showed independent performance for survival prognosis, clinic pathological features, immune infiltration, and drug sensitivity. Multivariate Cox analysis findings revealed that the risk scores derived from predictive models is independent prognostic markers for pancreatic patients. In conclusion, based on neutrophil marker genes, this research created a molecular typing and prognostic grading system for pancreatic cancer, this system was very accurate in predicting the prognosis, tumor immune microenvironment status, and pharmacological treatment responsiveness of pancreatic cancer patients.

## INTRODUCTION

By the year 2025, pancreatic cancer (PC) is estimated to become the third most significant contributor to cancer-related deaths in Europe, responsible for 4.7% of all cancer-related fatalities in humans [1]. Pancreatic ductal adenocarcinoma (PDAC) stands as the prevailing form of pancreatic cancer, carrying a heightened risk for individuals afflicted with diabetes or chronic pancreatitis [2, 3]. PDAC is a formidable malignancy with a significant mortality rate, with a mere 18% of patients surviving at the 1-year mark across all stages of the disease. It is difficult to detect the early subclinical symptoms of PDAC, such as lack of appetite, stomach discomfort, and back pain. Hence, the majority of patients with PDAC are identified in the intermediate and advanced stages with distant metastases [4–6]. In addition, PDAC is one of the cancer forms with the highest immunotherapy resistance [7]. The majority of individuals with PDAC do not react well to immunotherapy alone, immune checkpoint suppression treatment is unsuccessful for these individuals [8]. Although surgery is currently the only curative therapy for PC, even patients who undergo surgery have a high risk of cancer recurrence and may require additional treatment [9].

To improve the prognosis of PC patients, it is necessary to find new biomarkers for clinical diagnosis and prediction of therapeutic success [10]. PC recurrence and metastasis are significantly influenced by the immunological microenvironment. Recent investigations on the tumor microenvironment (TME) have underlined the significance of immune cell infiltration in PC development, metastasis, and immune evasion [11–14]. Mlecnik B. et al. earlier constructed an immunoscore-based cancer classification system for colon cancer and demonstrated that immune cell invasion has a greater predictive value than traditional tumor invasion (TNM stage) [15]. The implementation of immunological scoring systems will thus bring unique insights into the clinical diagnosis of PC and the prediction of therapeutic success.

Neutrophils, which are produced from hematopoietic stem cells in bone marrow, are plentiful in the bloodstream and a vital component of the innate immune system. In addition, neutrophils react efficiently to infection and inflammatory damage. Neutrophils represent a significant fraction of immune cells that invade various forms of cancer, including colorectal cancer, breast cancer, melanoma, and renal cell carcinoma [16–18]. Several tumor-secreting agents may reportedly attract neutrophils into the TME. CXCL1, CXCL2, CXCL5, and CXCL8 interact to the neutrophil surface receptors CXCR1 and CXCR2 to induce chemotaxis [19–21]. In addition, CD4+ T cells in the TME and the cytokine IL17 released by T cells contribute to the recruitment of neutrophils [22]. Neutrophils recruited into the TME interact with other TME components, which may restrict or promote tumor development. Consequently, neutrophils may be separated into two polarization states: N1 neutrophils, which limit tumor growth, and N2 neutrophils, which promote tumor development. The IFN- signaling pathway stimulates the production of IL12, ICAM1, and tumor necrosis factor (TNF), all of which are deadly to tumor cells and suppress tumor development. Transforming growth factor (TGF) promotes carcinogenesis by secreting arginase, MMP9, VEGF, and other chemokines to stimulate tumor angiogenesis [23–27].

PC’s aggressive traits are linked to its high fibroblast content and inflammatory TME including numerous immune cells, including neutrophils [28]. Experiments with animal models revealed that neutrophils interact with pancreatic stellate cells, tumor-associated macrophages, and extracellular matrix in the TME to significantly accelerate tumor growth [29–31]. Yet, neither the underlying processes nor the therapeutic uses of neutrophils have been explained. In order to offer a scientific foundation for clinical decision-making and risk management in patients, the purpose of this work was to examine the molecular mechanism of neutrophils in PC development and to identify neutrophil-related genes (NRGs) to design survival predictive models.

## MATERIALS AND METHODS

### Data acquisition and preprocessing

Single-cell sequencing data of PC were acquired from the Gene Expression Omnibus (GEO) database (https://portal.gdc.cancer.gov/) (registration number: GSE111672) [32]. The transcriptome data of PC tissues (data from 178 pancreatic tumor tissues and 4 paracancerous tissues) as well as associated clinical data (data from 185 PC samples) were obtained from The Cancer Genome Atlas (TCGA) database (https://portal.gdc.cancer.gov/) [33]. The GSE28735 [34, 35] (data from 45 PC tissues), GSE62452 [36] (data from 69 PC tissues), GSE57495 [37] (data from 63 PC tissues), and GSE85916 datasets (data from 80 PC tissues) were obtained from the GEO database. The “SVA” R package was used to remove batch effects between different datasets [38, 39]. Data in which the survival time of patients was less than 30 days were excluded and not included in the survival analysis.

### Single-cell data analysis and identification of NRGs

For quality control, analysis, and exploration of single-cell RNA expression data, the R software packages “Seurat” and “SingleR” were employed [40, 41]. To obtain high-quality data on single-cell RNA expression, the following filtering criteria were established: (1) exclusion of genes smaller than those detected in 3 cells, (2) exclusion of cells with fewer than 50 detected genes, and (3) exclusion of cells in which mitochondrial gene expression accounts for more than 5% of total gene expression. Single-cell RNA expression data were normalized using the “Normalize Data” program. The “Find Variable Feature” tool was used to determine the top 1500 genes whose expression changed significantly. The “Run PCA” tool was used to conduct principal component analysis (PCA) on the top 1500 genes, and the *P*-value of each principal component was obtained. The top 15 main components were chosen and subjected to t-distributed stochastic neighbor embedding (t-SNE) cell clustering analysis. To identify marker genes, differentially expressed genes across distinct cell clusters were examined using the following criteria: adjusted *P*-value < 0.05 and |log2 (fold-change)| > 1. Annotation of cell clusters was conducted using reference data from Human Primary Cell Atlas for reference-based annotation [42].

### Gene Ontology (GO) and Kyoto Encyclopedia of Genes and Genomes (KEGG) enrichment analysis

The R packages “org.Hs.eg.db”, “clusterProfiler”, “enrichplot”, and “ggplot2” were used to conduct GO and KEGG function and pathway enrichment studies of differentially expressed NRGs with adjusted *P*-value < 0.05 as the filter criterion [43]. Biological processes, cellular components, and molecular functions were the GO words.

### Consensus cluster analysis of NRGs

Using the “ConsensusClusterPlus” R software, data from the TCGA, GSE28735, GSE62452, GSE57495, and GSE85916 datasets were pooled to cluster all patients based on the expression of NRGs [44]. Based on the consensus matrix and cumulative distribution function (CDF) curves, the optimal number of clusters was determined [45]. Using the Kaplan-Meier technique and the log-rank test, the survival time between various groups was compared. Typing consistency was shown using a PCA with a strong capacity for dimension reduction [46].

### Differential expression analysis and typing analysis

Using the criterion |log2 (FC)| > 1 and adjusted *P*-value < 0.05, the “limma” R program was used to find differentially expressed genes across NRGcluster [47]. Differentially expressed genes were analyzed for GO and KEGG enrichment using the “org.Hs.eg.db”, “clusterProfiler”, “enrichplot”, and “ggplot2” R packages [43]. To assess further the influence of differently expressed genes on PC prognosis, the “ConsensusClusterPlus” R program was used to cluster all patients according to differentially expressed genes [44]. Using the Kaplan-Meier technique and the log-rank test, the survival time between various kinds was compared [48].

### Construction and validation of the prognostic risk score model

To further investigate the predictive usefulness of NRGs in PC and quantify the NRG score in each patient, univariate Cox regression analysis was used to screen NRGs linked with patient outcomes. Random survival forests-variable hunting (RSFVH) was used to screen and filter genes [49, 50]. A prognostic risk score model for PC was developed using a multivariable Cox regression analysis. The *P*-value of the findings of the survival analysis was utilized to test for the optimal gene combination or final prognostic risk score model. Data from TCGA datasets used as training sets, whilst GEO datasets GSE28735, GSE62454, GSE57495 and GSE85916 served as validation sets. Based on gene expression profiles and coefficients of Cox analysis, the risk score for each sample may be computed. Each sample was classified as either high-risk or low-risk based on a comparison to the training set’s median risk score.

### Correlation and independent prognostic analysis of clinical pathological features

Clinical data from the TCGA datasets and risk scores were combined and classified according to their clinical pathological characteristics. The Wilcoxon signed-rank test was used to assess risk score differences across groups. In addition, univariate and multivariate Cox regression model studies were conducted to determine if risk scores were independent of the clinic pathological characteristics of PC as predictive risk factors.

### Gene set variation analysis (GSVA) and gene set expression analysis (GSEA)

GSVA is a non-parametric, unsupervised technique that calculates the enrichment percentage of a certain gene set in each sample without requiring previous categorization of data [51]. In expression datasets, GSVA is often used to quantify changes in metabolic pathway and bioprocess activity. GSEA is a computer tool that determines if a certain gene set differs substantially between two biological states. In expression datasets, GSEA is often used to evaluate changes in pathway and bioprocess activity [52]. GSVA was done using the “GSVA” R package to compare the biological processes and metabolic pathways across patients with various subtypes and different risk categories [51, 53]. The “limma”, “org.Hs.eg.db”, “clusterProfiler”, and “enrichplot” R packages were used to run GSEA [43, 47, 52].

### Immunoassays

To investigate the variations in immune cell infiltration across patients belonging to distinct subtypes or risk groups, the “GSVA” and “GSEABase” R packages were used to determine the immune cell invasion score for each sample using ssGSEA [53, 54]. Based on the results of ssGSEA analysis, the level of immune cell invasion was compared between patients with PC belonging to different subtypes or risk groups. Additionally, the proportions of 22 immune cell subtypes, including seven T cell subtypes, naïve and memory B cells, plasma cells, natural killer (NK) cells, and bone marrow subsets, were calculated for each sample using the CIBERSORT algorithm [55]. *P*-value < 0.05 suggested that CIBERSORT accurately estimated the fraction of 22 immune cell isotypes. These samples were utilized to analyze the link between risk ratings and immune cell infiltration in further detail.

### Drug sensitivity analysis

“OncoPredict” is an R package for predicting drug response *in vivo* or in patients with cancer from cell line screening data [56]. To examine the responsiveness of PC patients to pharmacological treatment, the “OncoPredict” R program was used to evaluate variations in drug sensitivity across patients with distinct subtypes or risk groups [56].

### Gene expression analysis

The Gene Expression Profiling Interactive Analysis (GEPIA) database is an online resource for analyzing genetic differences, relationships, and survival using TCGA and Genotype-Tissue Expression (GTEx) data [57]. At the RNA level, the differential expression of the model genes LDHA, IL1R2, and TM4SF1 between tumor and no tumor tissues was assessed. The Human Protein Atlas (HPA) database (https://www.proteinatlas.org/), a public database designed to create maps of protein expression patterns in healthy cells, tissues, and cancer [58–60], was used to analyze the differential expression of LDHA, IL1R2, and TM4SF1 between tumor and non-tumor tissues at the protein levels.

### Data analysis

Using R software, data analysis and visualization were conducted (Ver 4.1.2). For regularly distributed parameters, the *t*-test was used to compare the means of two groups. We compared the means of two groups for non-normally distributed parameters using Wilcoxon rank sum tests. Using either the Spearman correlation analysis or the Pearson test, correlation was assessed. Using the Kaplan-Meier technique, the survival curve of several groups was produced. Using the log-rank test, the survival rates of several groups were compared. At *P*-value < 0.05, differences were deemed significant.

### Availability of data and materials

The datasets analyzed in this work may be found in the Supplementary Materials or contact with the first author.

## RESULTS

### Identification of neutrophil marker gene expression profiles

**Figure 1:**
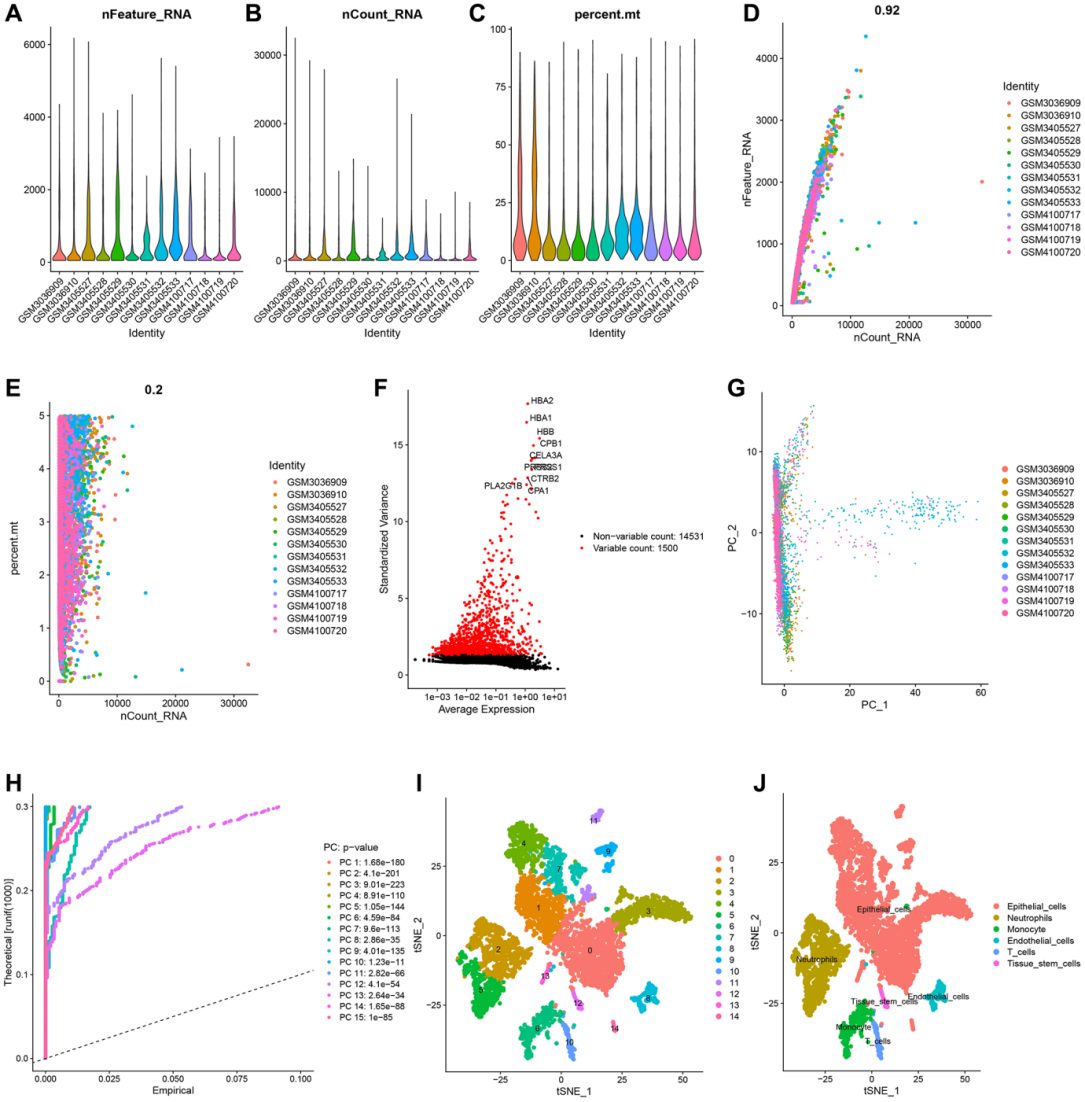
**Single-cell analysis and screening of neutrophils-related genes (NRGs).** (**A**) A visualization showing the number of genes in each cell as a violin. (**B**) A violin plot showing the total of each cell’s gene expression levels. (**C**) Violin plot showing each cell’s proportion of mitochondrial genes. (**D**) Scatterplot of the total gene expression levels and the number of genes present in each cell. (**E**) Scatterplot comparing the total gene expression levels in each cell to the proportion of mitochondrial genes. (**F**) Genes that vary significantly between cells. Red dots denote 1500 genes whose expression levels have changed significantly. The top ten most variable genes are labeled in the graph. (**G**) The cell distribution in the PC1 and PC2 dimensions. (**H**) Principal component analysis identified the top 15 principal components with a *P*-value < 0.05. (**I**) The study of t-Distributed stochastic neighbor embedding (t-SNE) identified 15 cell clusters. (**J**) Using maker genes, cell subtypes were further annotated and labelled.

**Figure 2:**
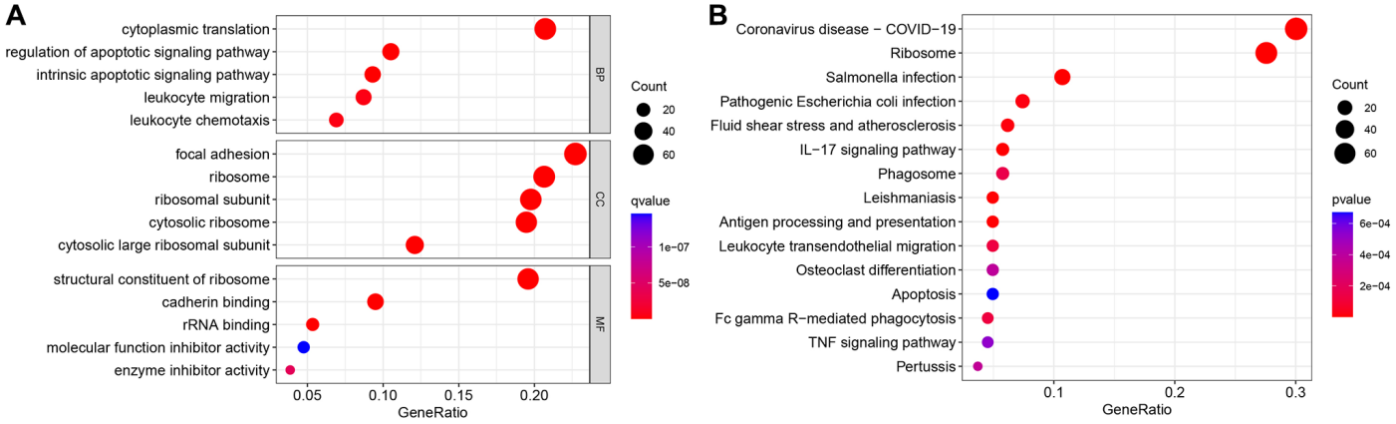
**Gene Ontology (GO) and Kyoto Encyclopedia of Genes and Genomes (KEGG) analyses.** (**A**) GO enrichment analysis. (**B**) KEGG enrichment analysis.

The GSE111672 dataset was obtained, which contains the RNA expression data of 27,000 cells from three individuals with PC. The range of genes discovered in each sample, the sequencing depth, and the proportion of mitochondrial content are shown in Figure 1A–1C, respectively. Single cells with a total number of identified genes more than 50, mitochondrial gene expression of less than 5%, and genes found in less than three cells were chosen (Figure 1A–1C). As seen in Figure 1D, 1E, sequencing depth was favorably connected with gene number and mitochondrial content, with correlation values of 0.92 and 0.2, respectively. After this, ten of the original 1500 highly variable genes between cells were labeled (Figure 1F). PCA was used to illustrate the top 15 principal components with *P*-value < 0.05 for the leading 1500 genes (Figure 1G, 1H). On the basis of the PCA findings, t-SNE analysis was done to further categorize all cells into 15 groups (Figure 1I). Each cell cluster was annotated using Human Primary Cell Atlas reference data. In clusters 2 and 5, neutrophils were found (Figure 1J). 350 NRGs were identified in total (Supplementary Table 1). GO functional enrichment and KEGG pathway enrichment studies were then used to study the biological function of these marker genes. Neutrophil marker genes were mostly engaged in biological processes such as leukocyte mobility, leukocyte chemotaxis, ribosomes, and plaque formation, as shown by GO functional enrichment analysis (Figure 2A). KEGG pathway enrichment analysis showed that these genes were largely associated with COVID-19, ribosomes, leukocyte trans endothelial migration, IL17 signaling pathway, TNF signaling route, and apoptosis (Figure 2B).

### Classification and characteristics based on the expression characteristics of neutrophil marker genes

**Figure 3:**
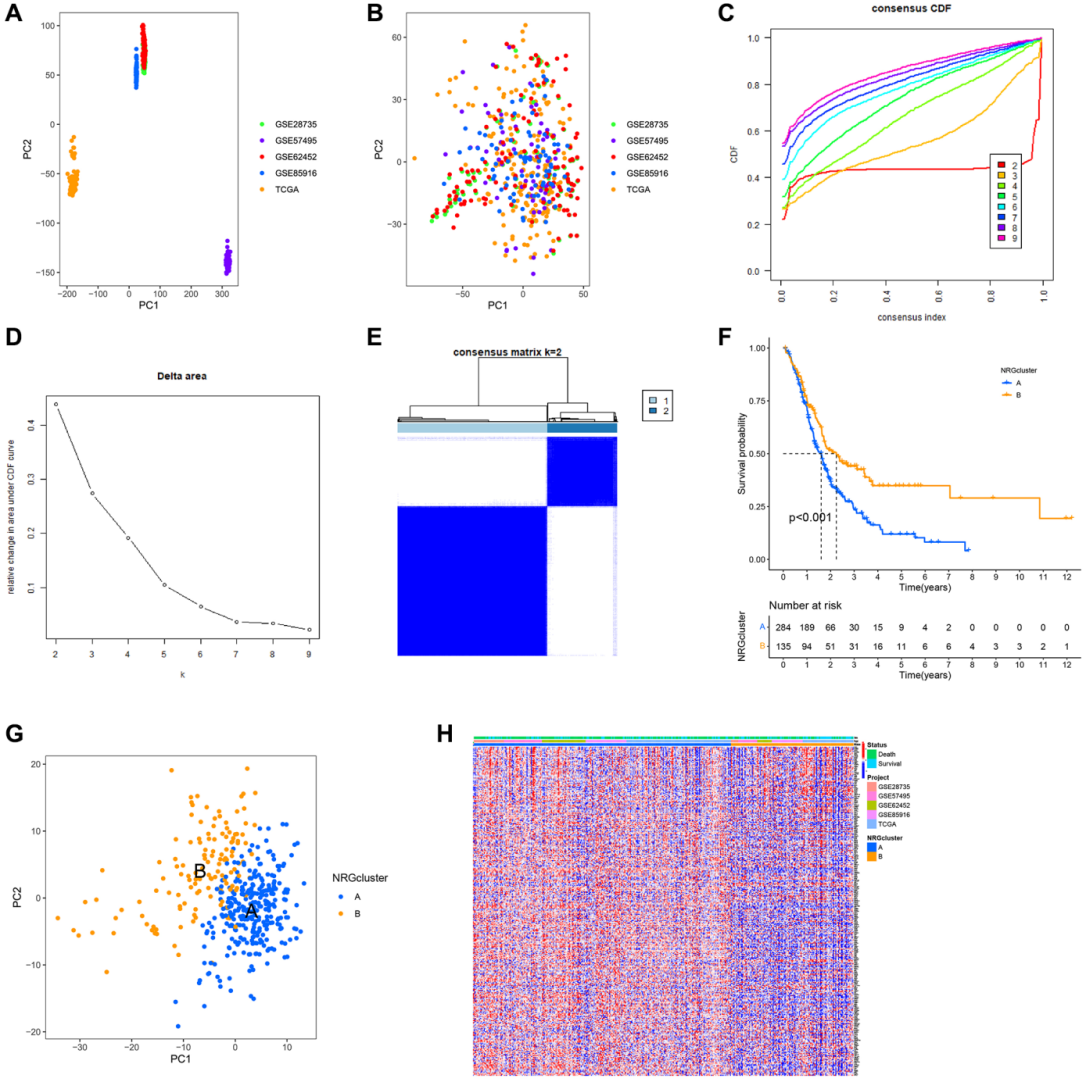
**Establishment of neutrophils-related genes (NRGs) cluster subtypes.** (**A**) Principal component analysis (PCA) plot of the overall expression of all samples before batching. (**B**) PCA plot of the overall expression of all samples after batching. (**C**–**E**) Process and results of consistency cluster analysis, which classified the data into NRGclusters A and B. (**F**) Survival curves of patients with NRGclusters A and B. The prognosis of patients with NRGcluster B was significantly better than that of patients with NRGcluster A. (**G**) PCA plot of NRGclusters A and B. (**H**) The expression of neutrophil-associated genes in NRGclusters A and B, most neutrophil-associated genes are upregulated in NRGcluster A.

Using PCA, the TCGA, GSE28735, GSE62452, GSE57495 and GSE85916 datasets were combined to correct the data. After adjustment, the batch effect was effectively eliminated (Figure 3A, 3B). According to the expression of NRGs, the samples from GSE28735, GSE57495, GSE62452, GSE8516 and TCGA data sets were identified as two immune subtypes, NRGcluster A and NRGcluster B, which respectively represent high and low expression of NRGs. The k value should be adjusted to 2 for best performance, as evidenced by the area under the CDF curve and its relative change (Figure 3C–3E). In Figure 3C, when the slope of the curve in the CDF diagram is the most gentle, the clustering analysis results are the most reliable, and the corresponding k value is the best k value; Figure 3D shows the relative change of area under the CDF curve compared to k and k-1, and since there is no k = 1, the first point represents the total area under the CDF curve for k = 2; Figure 3E shows the matrix heat map when k = 2, and the white part rarely has blue infection, indicated that there is less interference between subgroups. To sum up, k = 2 is the optimal k value, and when k is 3 or other values, the interference between subgroups cannot be minimized. The Kaplan-Meir survival analysis indicated that patients with the NRGcluster A subtype had a poorer survival rate than those with the NRGcluster B subtype (*p* < 0.001) (Figure 3F). The PCA findings indicated a batch effect between NRGcluster A and B, suggesting that both clusters had strong internal consistency (Figure 3G). The survival and mortality status of the two patient groups were shown as heat maps in Figure 3H, along with the expression of neutrophil marker genes such as SRGN, BCL2A1, ALOX5AP, FPR1, BASP1, and CSFR. Individuals with the NRGcluster A subtype demonstrated elevated expression levels of neutrophil marker genes, suggesting that these patients may have aggressive neutrophil infiltration (Figure 3H).

**Figure 4:**
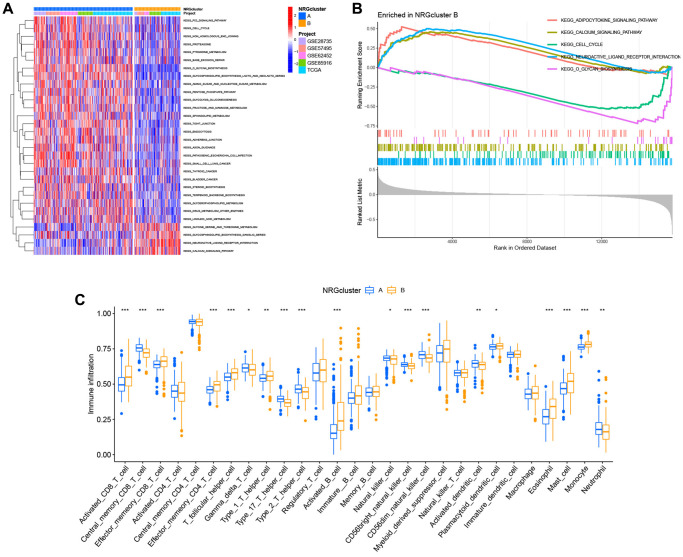
**Analysis of metabolic pathways and immunological infiltration for subtypes of the neutrophil-related genes (NRGs) cluster.** (**A**) Gene set variation analysis (GSVA) results. (**B**) Gene set expression analysis (GSEA) results. (**C**) Single-sample GSEA (ssGSEA) results.

Subsequently, the unique biological processes and metabolic pathways of patients with distinct subtypes were investigated. The heat map generated by GSVA indicated substantial variations between the two groups in the activation and inhibition of 30 metabolic pathways. P53 signaling pathway, cell cycle, base excision repair, amino sugar and nucleotide sugar metabolism, glycolysis and gluconeogenesis, sphingolipid metabolism, endocytosis, glycerophospholipid metabolism, and linoleic acid metabolism were highly abundant in NRGcluster A. In contrast, NRGcluster B was markedly enriched in glycine, serine, and threonine metabolism, neuroactive ligand-receptor interaction, and calcium signaling pathway (Figure 4A). GSEA demonstrated that the neuroactive ligand-receptor interaction, the calcium signaling system, and the adipocytokine signaling pathway were significantly enriched in NRGcluster B patients. The cell cycle and O-glycan production were dramatically enhanced in NRGcluster A patients (Figure 4B).

We also assessed the TME status of the two subtypes using ssGSEA. Immune cell infiltration differed dramatically across subtypes. Neutrophils, central memory CD8+ T cells, Th1 cells, Th17 cells, and eosinophils were the most abundant infiltrating cells in NRGcluster A, while activated CD8+ T cells were the least abundant (Figure 4C).

**Figure 5:**
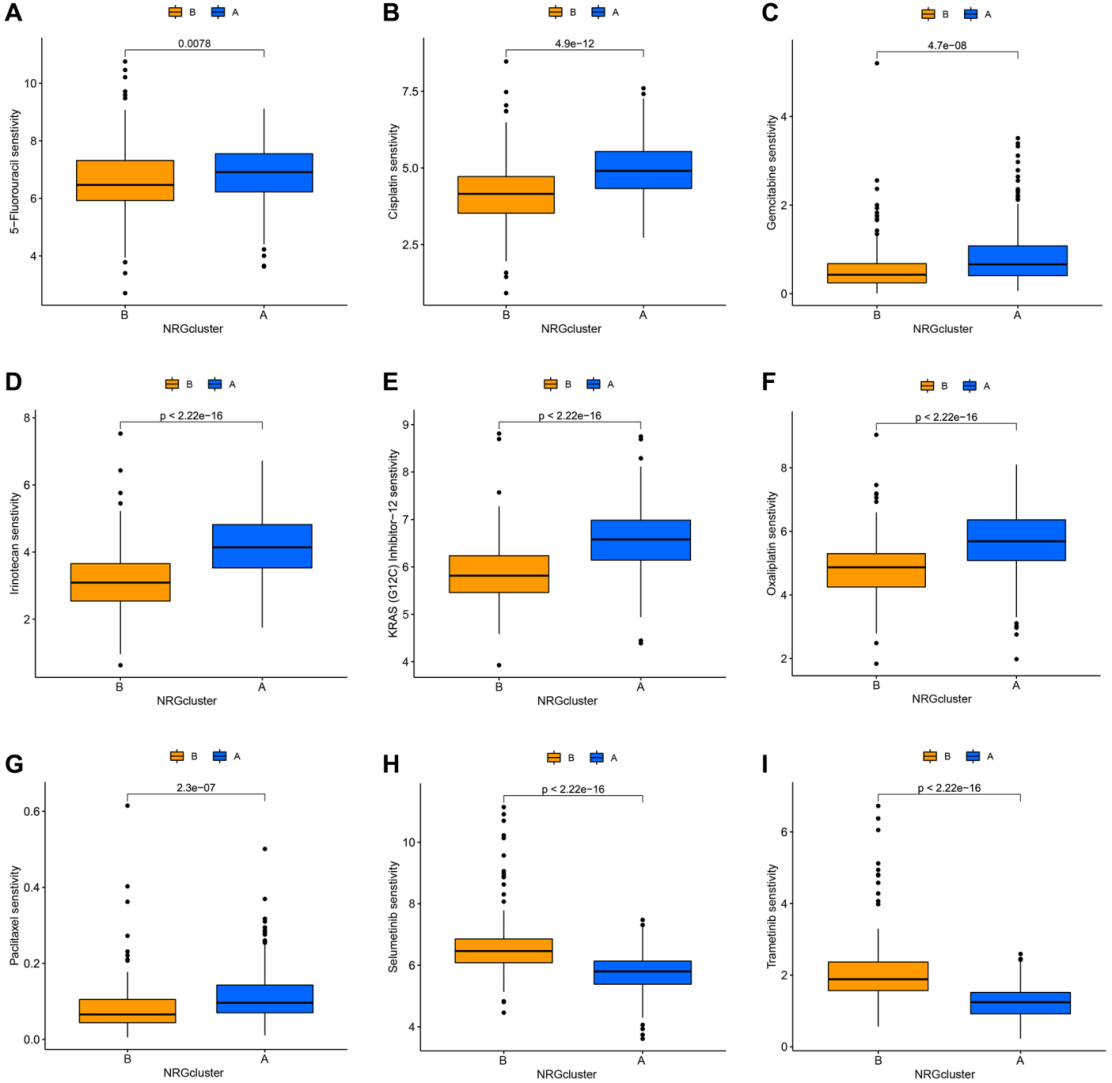
**Correlation between neutrophil-related genes (NRGs) cluster subtypes and drug sensitivity.** Increased sensitivity of NRGcluster B to 5-fluorouracil (**A**), cisplatin (**B**), KRAS (G12C) inhibitor-12 (**C**), gemcitabine (**D**), irinotecan (**E**), oxaliplatin (**F**), and paclitaxel (**G**), increased sensitivity of NRGcluster A to selumetinib (**H**) and trametinib (**I**).

The Genetics of Drug Sensitivity in Cancer database was used to evaluate the relationship between the two categories and the sensitivity to numerous first-line chemotherapy treatments. The NRGcluster B subtype was more sensitive to 5-fluorouracil, cisplatin, KRAS (G12C) inhibitor-12, gemcitabine, irinotecan, oxaliplatin, and paclitaxel, but less sensitive to selumetinib and trametinib (Figure 5A–5I).

### Genotyping based on differentially expressed genes

**Figure 6:**
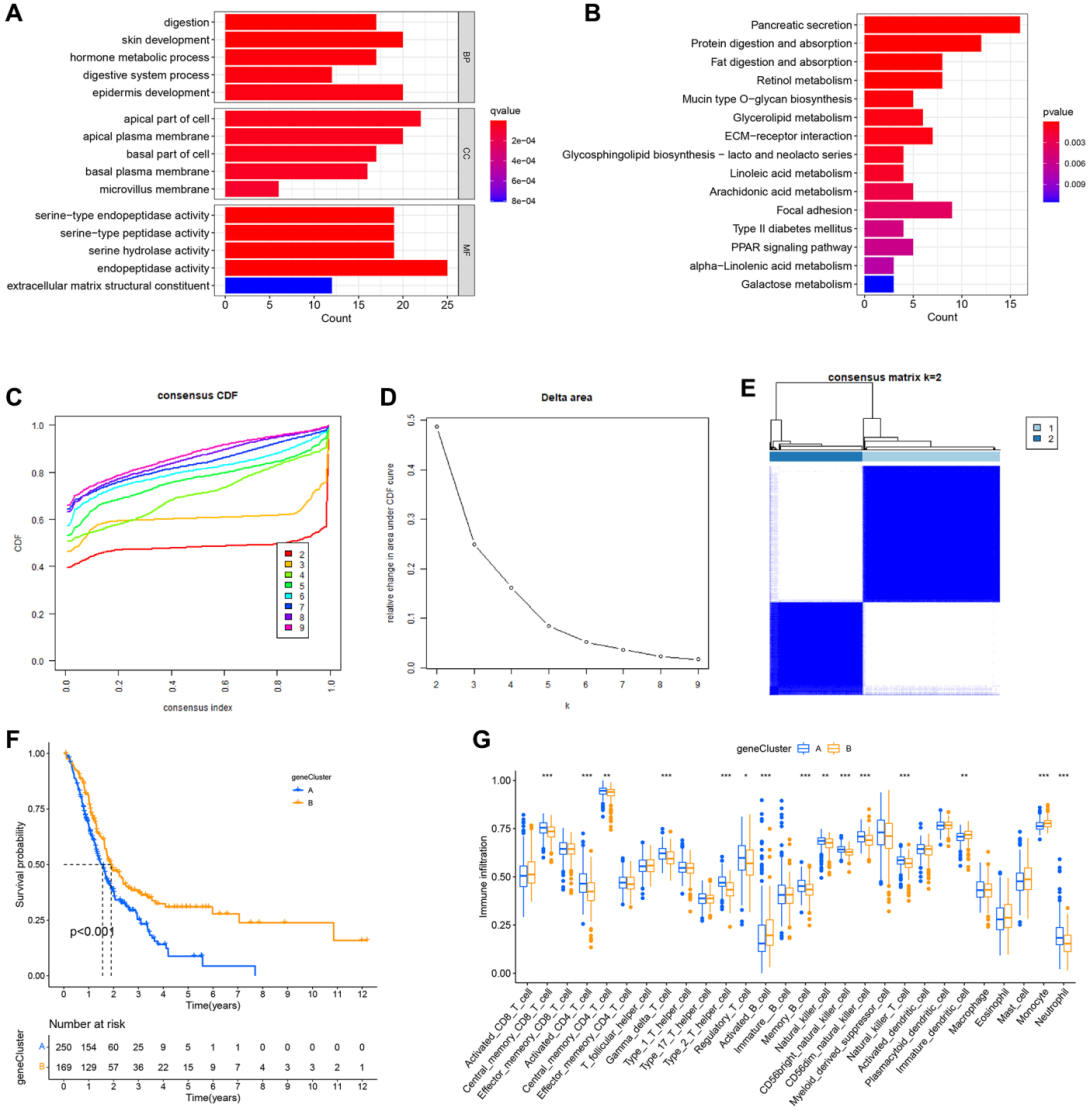
**Formation of subtypes of gene clusters.** (**A**) Analysis of Gene Ontology (GO) enrichment. (**B**) Enrichment analysis of the Kyoto Encyclopedia of Genes and Genomes (KEGG). (**C**–**E**) The methodology and outcomes of consistency cluster analysis, which categorized the data into geneClusters A and B. (**F**) The survival curves for patients with the A and B geneClusters. Patients with geneCluster B had a much better prognosis than those with geneCluster A. (**G**) Single-sample gene set enrichment analysis (ssGSEA) findings.

Subsequently, the genetic alterations and underlying processes of NRG expression patterns were investigated. Using the “limma” R package, the differentially expressed genes of NRGcluster A and B were selected based on the following criteria: |log2 FC| > 1 and adjusted *P*-value < 0.05. A total of 285 genes with differential expression were found (Supplementary Table 2). The functions of these differentially expressed genes were then identified. The differentially expressed genes were mostly connected with epidermal development, the apical region of the cell, endopeptidase activity, extracellular matrix structural component, etc., as determined by GO functional enrichment analysis (Figure 6A). The majority of differentially expressed genes were involved in pancreatic secretion, protein digesting, and absorption-related processes, as shown by KEGG pathway enrichment analysis (Figure 6B). To examine further the influence of differently expressed genes on PC prognosis and tumor immune microenvironment, all patients were grouped using the “Consensus Cluster Plus” R program based on differentially expressed genes. Two immunological isotypes (geneClusters A and B) were distinguished (Figure 6C–6E). Patients with geneCluster A had a considerably shorter survival rate than those with geneCluster B (Figure 6F). The ssGSEA found distinct immune cell infiltration between the two categories. Neutrophils, activated CD4+ T cells, and central memory CD8+ T cells were the most abundant infiltrating cells in geneCluster A, whereas monocytes and activated CD8+ T cells were less abundant (Figure 6G).

### Establishment of the PC prognostic risk model based on NRGs

**Figure 7:**
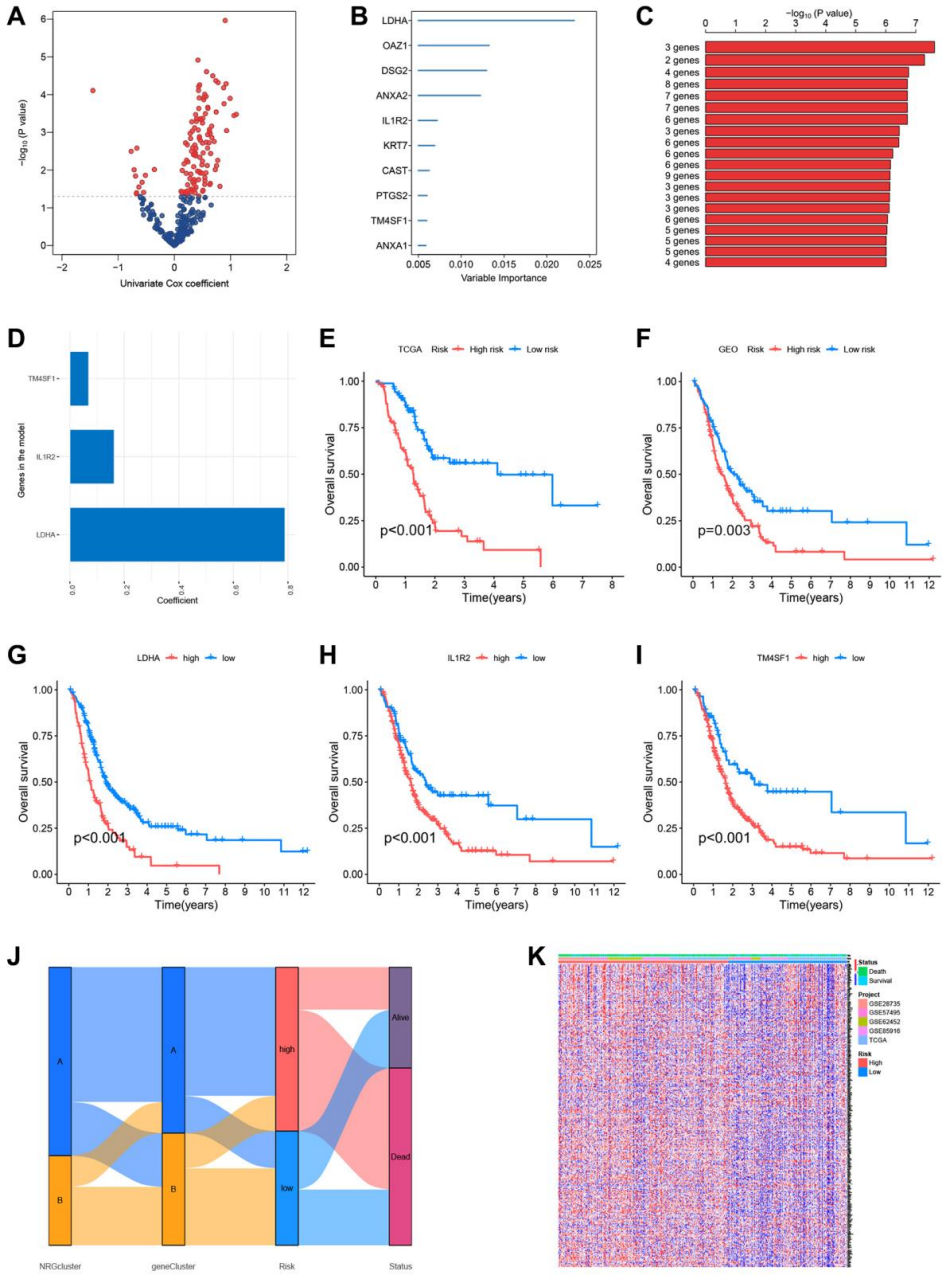
**Construction of the prognostic risk score model.** (**A**) Volcano plot of univariate Cox regression analysis results. Red dots represent neutrophil-related genes associated with pancreatic cancer prognosis. (**B**) Random survival forest analysis identified the top 10 important genes. (**C**) After survival analysis of 2^10^−1 = 1023 combinations, the top 20 models were sorted according to *P*-values. The best model comprised three genes. (**D**) Coefficients of genes in the model. (**E**) The Cancer Genome Atlas (TCGA) dataset (Kaplan-Meir curve of the training set). (**F**) GSE28735, GSE62452, GSE57495, and GSE85916 datasets from the Gene Expression Omnibus (GEO) platform (Kaplan-Meir curves of the validation set). Survival curves of patients according to the expression of LDHA (**G**), IL1R2 (**H**), and TM4SF1 (**I**). (**J**) Sankey plots of different subtypes, risk groups, and survival states. (**K**) The expression of neutrophil-associated genes in high-risk and low-risk groups, most neutrophil-associated genes are upregulated in the high-risk groups.

To further study the predictive usefulness of NRGs in PC, a univariate Cox regression analysis was conducted to identify NRGs linked with patient prognosis. Each potential gene is represented by a red dot in the volcano map (Figure 7A). Using the RSFVH method, the 10 most significant genes were analyzed further (Figure 7B). Using an algorithm, survival analysis *p*-values were analyzed. The optimal gene combination (LDHA, IL1R2, and TM4SF1) was determined (Figure 7C), and a predictive model was created using multivariate Cox regression. The correlation coefficients of the three model genes were shown in Figure 7D, and risk scores of each sample were computed with the help of Cox coefficients and gene expression profiles. The GEO database datasets GSE28735, GSE62452, GSE57495, and GSE85916 were utilized as the validation set. To evaluate the accuracy of the prognostic model, the training and validation sets were split into high-risk and low-risk groups based on the median risk score. Survival study of the TCGA and GEO datasets found that the survival time of high-risk patients was shorter than that of low-risk patients (Figure 7E, 7F). For all three model genes (LDHA, IL1R2, and TM4SF1), the high-expression group had a lower life period than the low-expression group (Figure 7G–7I). The alluvial plot indicated the link between NRGcluster typing, gene Cluster type, risk categorization, and sample survival status (survival or death) and supported the reliability and consistency of the analytical findings (Figure 7J). The majority of genes were elevated in the high-risk group, indicating that high-risk individuals may have aggressive neutrophil infiltration (Figure 7K).

### Correlation between risk model and clinical pathological features

**Figure 8:**
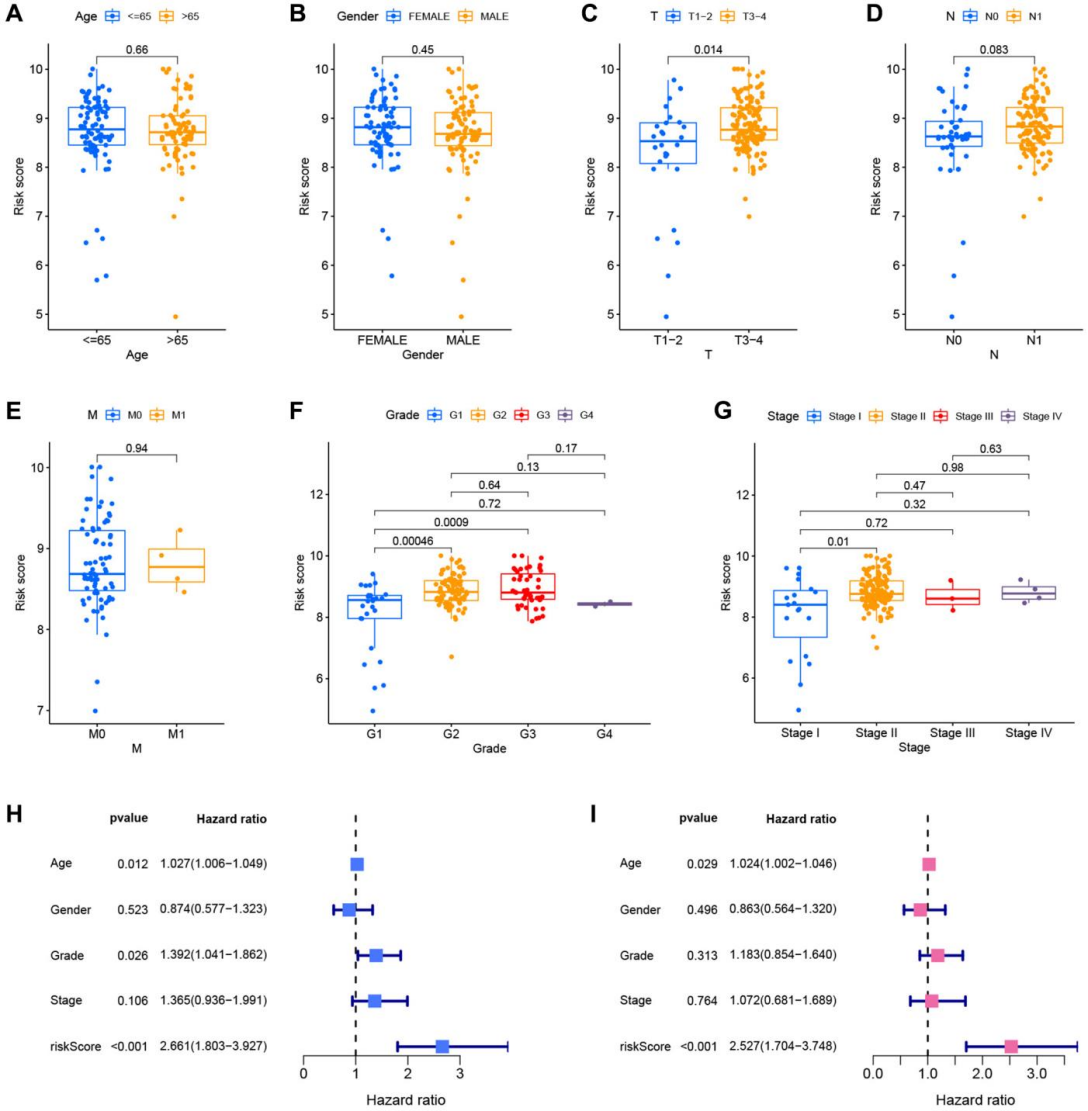
**Correlation and independent prognostic analysis of clinical pathological features.** Differential risk scores between age groups (**A**), gender groups (**B**), different T stages (**C**), different N stages (**D**), different M stages (**E**), different pathological grades (**F**), and different clinical stages (**G**). Forest plots of univariate Cox analysis (**H**) and multivariate Cox analysis results (**I**).

After merging clinical data and risk scores from TCGA datasets, the risk scores were compared across clinical groups using the Wilcoxon signed-rank test to investigate the association between the model and the clinical features of the patients. The risk ratings did not vary substantially across ages, genders, or M stages (Figure 8A, 8B and 8E). Due to the limited number of patients with M-stage tumors, the Wilcoxon signed-rank test findings might be distorted. The risk scores of patients with advanced T-stage, pathological grade, and clinical stage were considerably elevated (Figure 8C, 8F and 8G). The difference between N1 and N0 patients was close to statistical significance (*p* = 0.083), and stage N1 patients had a higher risk score (Figure 8D). Age, pathological grade, and risk score were substantially linked with the prognosis of patients with PC, as evidenced by the forest plot of univariate Cox analysis findings (Figure 8H). Furthermore, the forest plot of multivariate Cox analysis findings revealed that age and risk score are independent prognostic markers for PC patients (Figure 8I).

### Molecular pathway and immune infiltration analysis based on the prognostic risk model

**Figure 9:**
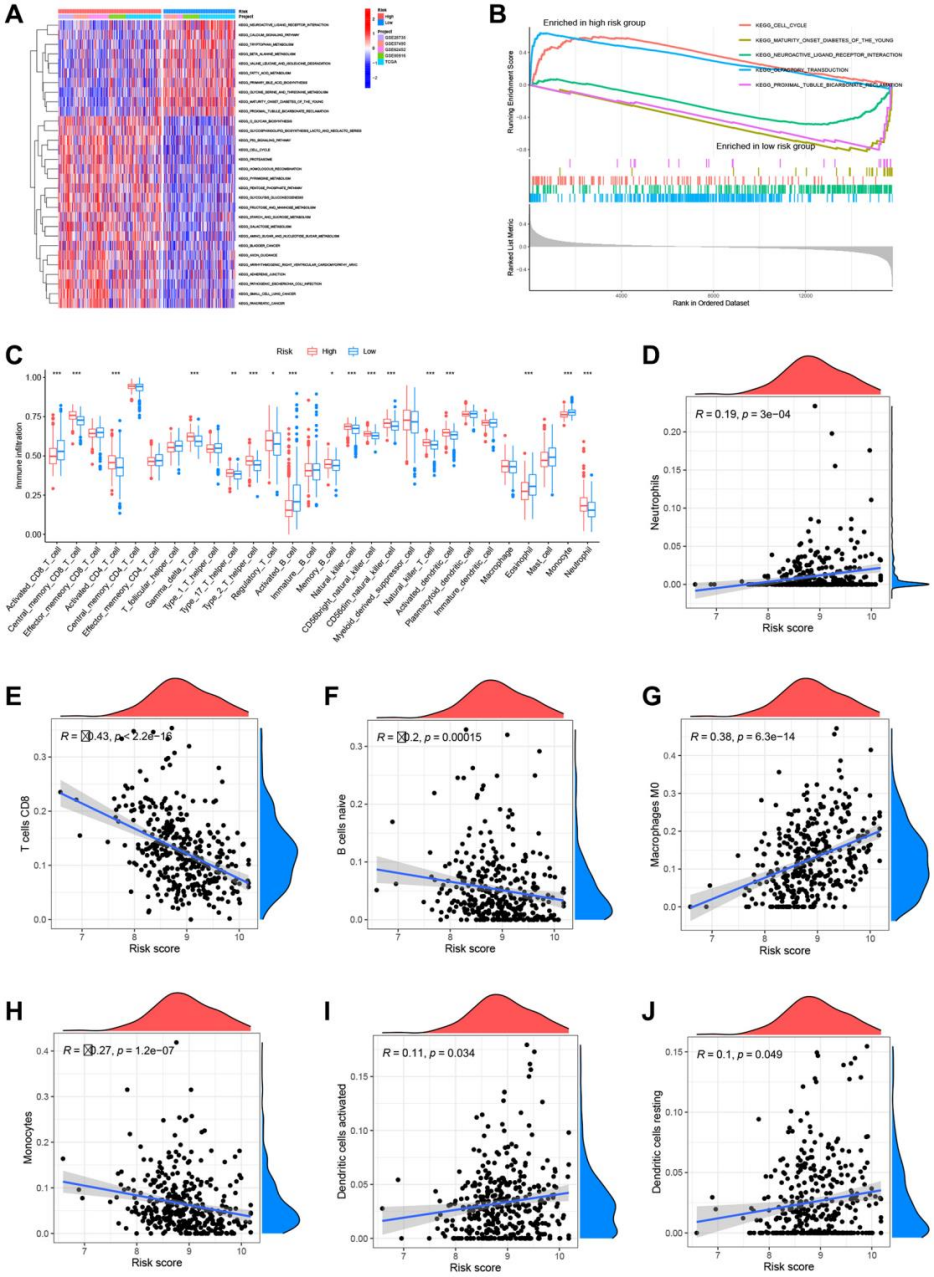
**Analysis of metabolic pathways and immune infiltration using the prognostic models.** (**A**) Gene set variation analysis (GSVA) results. (**B**) Gene set enrichment (GSEA) results. (**C**) Single-sample GSEA (ssGSEA) results. Risk scores were significantly and positively correlated with neutrophil infiltration levels (**D**), significantly and negatively correlated with CD8+ T cell infiltration levels (**E**), significantly and negatively correlated with naïve B cell infiltration levels (**F**), significantly and positively correlated with M0 macrophage infiltration levels (**G**), significantly and negatively correlated with monocyte infiltration levels (**H**), significantly and positively correlated with activated dendritic cell infiltration levels (**I**), and significantly and positively correlated with resting dendritic cell infiltration levels (**J**).

To assess the metabolic pathway activity in high-risk and low-risk samples, GSVA and GSEA were conducted. The high-risk group had considerably greater glycan biosynthesis, P53 signaling pathway, and cell cycle activity than the low-risk group. In contrast, the activities of neuroactive ligand-receptor interaction and calcium were increased in the group at low risk (Figure 9A). Cell cycle and olfactory transduction were typically active in the high-risk group, while maturity-onset diabetes of the young and neuroactive ligand-receptor interaction were passive (Figure 9B). To further study the link between risk score and TME in PC, ssGSEA was used to analyze immune cell infiltration across groups with high and low risk. The high-risk group had higher infiltration of activated CD4+ T cells, NK cells, and neutrophils, whereas the low-risk group demonstrated increased infiltration of activated CD8+ T cells and activated B cells (Figure 9C). In addition, the CIBERSORT method was used to calculate the infiltration scores of 22 immune cell subtypes in each sample (Supplementary Figure 1) (7 T cell subtypes, naïve and memory B cells, plasma cells, NK cells, bone marrow subsets, etc.). Neutrophils, M0 macrophages, active dendritic cells, and resting dendritic cells associated considerably and positively with the risk score, while CD8+ T cells, naïve B cells, and monocytes linked significantly and negatively with the risk score (Figure 9D–9J).

### Drug sensitivity analysis

**Figure 10:**
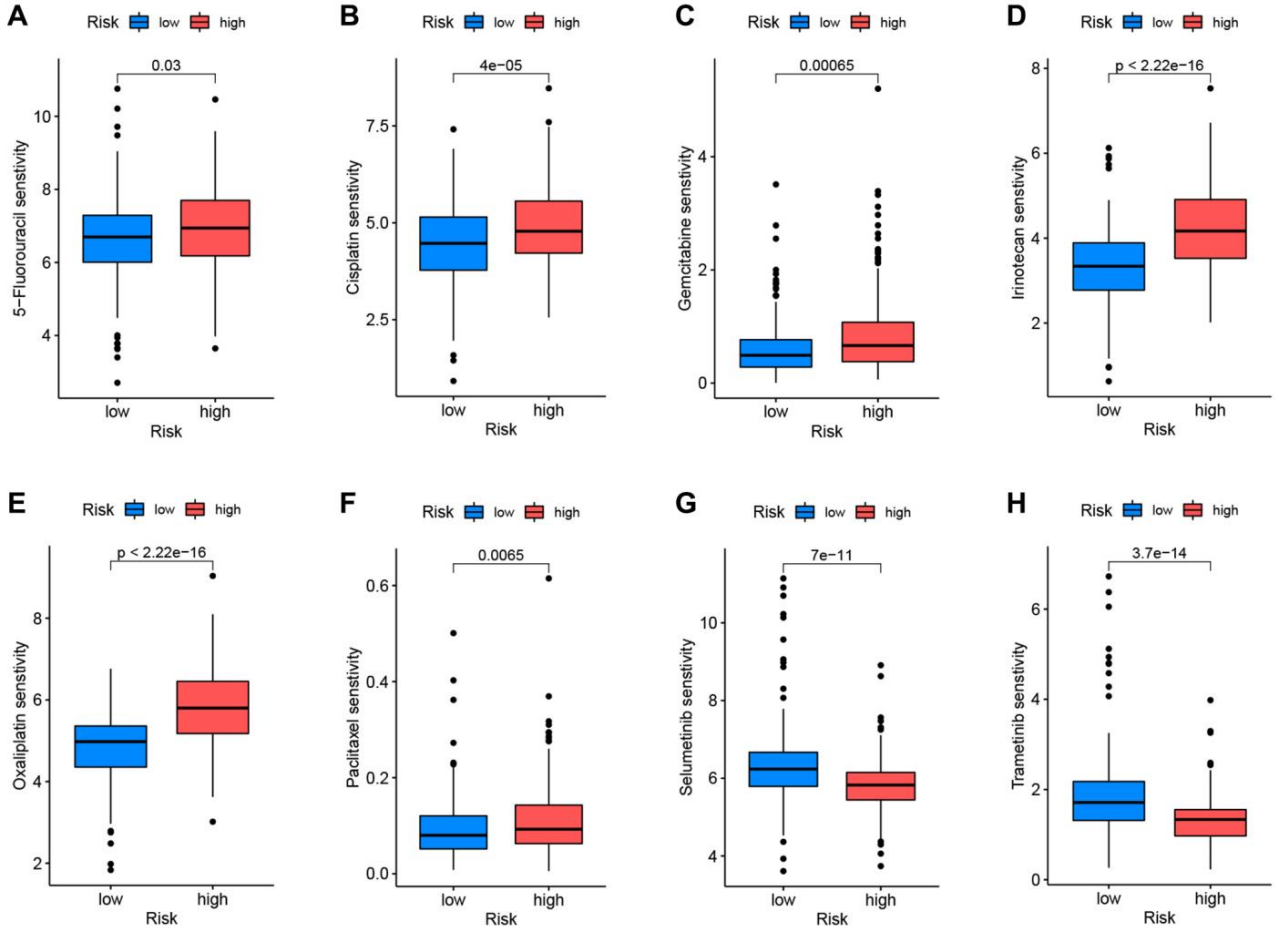
**Correlation between drug sensitivity and prognostic risk scores.** Low-risk patients were sensitive to 5-fluorouracil (**A**), cisplatin (**B**), gemcitabine (**C**), irinotecan (**D**), oxaliplatin (**E**), and paclitaxel (**F**). High-risk patients were sensitive to selumetinib (**G**) and trametinib (**H**).

The R program “oncoPredict” was used to examine the differential medication sensitivity between high-risk and low-risk groups. Low-risk patients were sensitive to 5-fluorouracil, cisplatin, gemcitabine, irinotecan, oxaliplatin, and paclitaxel, whereas high-risk patients were susceptible to selumetinib and trametinib (Figure 10A–10H). These results help influence clinical medication for PC patients.

### Gene expression analysis

**Figure 11:**
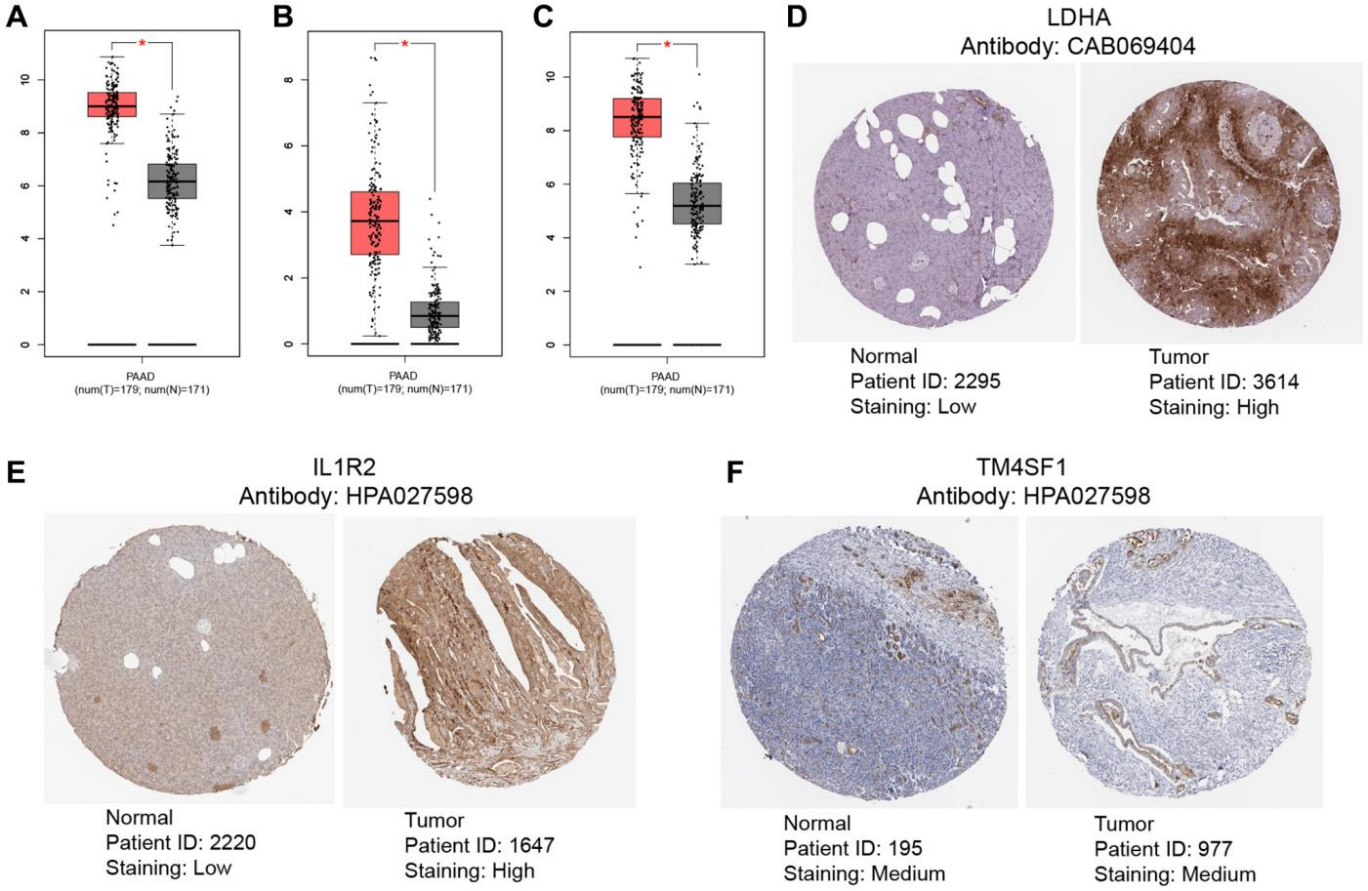
**Validation of levels of gene expression.** In pancreatic tumor tissues, the expression levels of LDHA (**A**), IL1R2 (**B**), and TM4SF1 (**C**) were considerably greater than in healthy tissues. Immunohistochemical examination indicated that the expression levels of LDHA (**D**) and IL1R2 (**E**) were considerably elevated in pancreatic tumor tissues compared to healthy tissues, TM4SF1 (**F**) was moderately expressed in both pancreatic tumor tissues compared to healthy tissues.

To evaluate the differential expression levels of model genes LDHA, IL1R2, and TM4SF1 across tumor and non-tumor tissues at the RNA level, scatter plots were generated using the GEPIA database. The expression of model genes was higher in tumor tissues compared to non-tumor tissues (Figure 11A–11C). The HPA database was queried for pictures of immunohistochemical investigation of LDHA, IL1R2, and TM4SF1. Figure 11D, 11E demonstrates that LDHA and IL1R2 were highly elevated in PC tissues, but TM4SF1 was moderately expressed in both PC and non-tumor tissues (Figure 11F).

## DISCUSSION

The current outlook for PC, an aggressive malignant tumor, is dismal. PDAC, the most common form of pancreatic cancer, is a highly aggressive and moderately resistant pancreatic exocrine tumor. Previous research has demonstrated that PDAC tumors exhibit intratumor heterogeneity, which contributes to the extensive metastasis observed at the time of diagnosis and poor prognosis and poses a significant challenge to medical professionals attempting to develop effective treatment strategies [61, 62]. In addition to imaging techniques (such as computed tomography and magnetic resonance imaging), clinics routinely utilize tumor markers (such as CEA and CA199) to diagnose PC. Nonetheless, the sensitivity and specificity of these indicators for diagnosing PC remain inadequate. In addition, these markers are not considerably elevated during the disease's early stages [63]. This research focused on TME, which directly contributes to the aggressiveness of PC, in order to discover precise biomarkers and effective targeted treatments and to comprehend the prospective features of PC. Neutrophils, which serve as the first line of defense against pathogen invasion, are a crucial component of the TME. In addition, neutrophils are essential for antitumor immunity. Following carcinogenesis, neutrophils recruited to the TME may facilitate interactions between tumor cells and other stromal cells, therefore influencing the direct or indirect development of the tumor [19, 22, 64]. Neutrophils may also promote the development of primary pancreatic tumors by producing neutrophil extracellular traps, therefore promoting cancer-associated hypercoagulability and accelerating the formation of metastatic lesions [65–67].

Prior research has showed that single-cell transcriptome analysis may determine cell type abundance using a large number of transcriptome-specific genes. In cancer research, advances in the creation and availability of single-transcriptome analysis have improved cancer detection and therapy [68–70]. This work merged several single-cell datasets, identified main cell subtypes (including T cells, neutrophils, monocytes, tissue stem cells, epithelial cells, and endothelial cells), and obtained neutrophil-specific marker genes. The neutrophils were then extensively tested to determine tumor characteristics. On the basis of univariate Cox regression analysis, prognosis-related neutrophil marker genes were evaluated. A prognostic risk model consisting of three important genes (LDHA, IL1R2, and TM4SF1) was developed. The model's independent prediction performance and connection with clinical pathological characteristics, degree of immune infiltration, and medication sensitivity were validated. Our results revealed that neutrophils play a crucial role in PC therapy responsiveness and prognosis.

Clustering facilitated the classification of PC patients from the TCGA, GSE28735, GSE62452, GSE57495, and GSE85916 datasets into NRGcluster A and NRGcluster B. The patient prognosis, characteristics of TME immune cell infiltration, biological behavior, and medication sensitivity differed dramatically between the two neutrophil clusters. The outcomes of GSVA and ssGSEA suggested that NRGcluster A was enriched in the cell cycle and cancer metabolic pathway and that neutrophil infiltration was discernable. Prior research and Kaplan-Meir curves revealed that the prognosis for patients with NRGcluster A type was bad. Subsequently, 285 genes with differential expression between NRGclusters were discovered. Similar to the clustering findings of neutrophil phenotypes, two genomic subgroups were established on the basis of distinctive genes highly linked with cell behavior and immunological activation. Results suggested that neutrophil activation is critical for the growth and prognosis of PC tumors.

Given the variety of NRGs, there is an urgent need to characterize NRG patterns in PC patients. This research thus devised a scoring system for measuring NRGs in PC patients. The survival prediction model included LDHA, IL1R2, and TM4SF1. Using the GSE28735, GSE62452, GSE57495, and GSE85916 datasets, the correctness of the model was validated. LDHA, which is increased in hematological malignancies and solid cancers, encodes the M-subunit of lactate dehydrogenase (LDH), which regulates aerobic glycolysis to fulfill the energy and biosynthetic demands of malignant tumors [71, 72]. According to reports, LDH overexpression is an independent poor prognostic marker in individuals with PC. Furthermore, in hypoxic circumstances, the upregulated LDH stimulates the production of LDHA in PDAC cells [73, 74]. LDH synthesis caused by LDHA boosts the aggressiveness and metastasis of PC and enhances resistance to radiation and chemotherapy [75, 76]. In renal cell carcinoma, LDHA promotes metastasis by stimulating epithelial-mesenchymal transformation, which can be inhibited by LDHA inhibitors [77]. IL1R2 is located on chromosome 2q12 and encodes IL1R2, which comprises 398 amino acids. In contrast to IL1R1, IL1R2 mainly acts as an endogenous inhibitor to prevent IL1 from binding to IL1R1 and inhibits IL1 signaling [78]. In renal cell carcinoma, IL1R2 promotes tumor progression through the JAK2/STAT3 pathway. The overexpression of IL1R2 reverses G1 phase inhibition and cell cycle arrest, promoting cancer cell proliferation, migration, and invasion [79]. Meanwhile, in colorectal cancer, IL1R2 is involved in activating IL6, VEGFA, and MEK/ERK, promoting cell proliferation and metastasis, promoting tumor angiogenesis, and enhancing tumor drug resistance [80]. IL1R2 is also reported to be involved in the progression of osteosarcoma, non-small cell lung cancer, liver cancer, and lymphoma [81–84]. TM4SF1, which is located on human chromosome 3, encodes a specific quadruple transmembrane low molecular weight glycoprotein. Previous studies have demonstrated that TM4SF1 promotes cell proliferation and survival through JAK2/STAT3 signaling and PI3K/AKT/mTOR-related signaling pathways. Additionally, TM4SF1 can promote cell migration, invasion, and stemness maintenance through the Wnt/β-catenin/c-Myc/SOX2 axis in various cancer, such as colorectal cancer [85–87]. One study reported that TM4SF1 was upregulated in glioma tumor tissues and cell lines and that TM4SF1 expression was inversely correlated with patient survival [88]. Additionally, the correlation between three key genes and the tumor immune microenvironment comprising neutrophils has also been explored in previous studies. For example, LDHA downregulation mediated by the PI3K/Akt-HIF-1α pathway results in glycolytic inhibition, which leads to neutrophil immunosuppression during sepsis [89]. In PDAC, the expression of IL1R2, encoding the bait receptor IL1R2, is associated with an increased abundance of Th2 cells and eosinophils [90]. Similar to CD63, TM4SF1 encodes transmembrane proteins that are also involved in signaling in immune cells, such as neutrophils [91].

According to these data, LDHA, IL1R2, and TM4SF1 are risk genes for PC, which is consistent with the findings of prior research. The high-risk group had a higher infiltration of activated CD4+ T cells, NK cells, and neutrophils, in addition to poor clinical pathological features and prognosis. Lastly, the GEPIA and HPA databases were consulted to validate the RNA and protein concentrations, respectively, that the model predicted.

In conclusion, based on the results of bioinformatics studies, we have revealed the molecular characteristics and prognostic role of neutrophils in pancreatic cancer, which will be helpful for the diagnosis and treatment of pancreatic cancer patients. However, the online database can only be used for the verification of early protein levels, the experimental results may not support the findings of computer analysis, and the role of neutrophils in the etiology of pancreatic cancer has not been determined, more experimental studies are needed to confirm the conclusions of this study. In addition, pancreatic cancer may present distant metastases *in vivo*, and the samples evaluated in this study did not include lung, bone, brain, and other types of metastases, which will be the subject of our future research. Therefore, more large-sample and multicenter studies will be necessary to verify the validity of our diagnostic and prognostic models.

## CONCLUSIONS

This research revealed an extensive variety of regulatory mechanisms used by NRGs in the TME in PC. Differences in NRG expression patterns contribute greatly to the variety and complexity of the TME in individuals with PC. The developed neutrophil-related molecular typing and prognostic models will facilitate the thorough assessment of individual patients with PC and enhance our comprehension of the cell infiltration features of the TME. This novel biomarker will lead the development of personalized and successful targeted therapies.

## Supplementary Materials

Supplementary Figure 1

Supplementary Table 1

Supplementary Table 2

## References

[1] Sung H, Ferlay J, Siegel RL, Laversanne M, Soerjomataram I, Jemal A, Bray F. Global Cancer Statistics 2020: GLOBOCAN Estimates of Incidence and Mortality Worldwide for 36 Cancers in 185 Countries. CA Cancer J Clin. 2021; 71:209–49. 10.3322/caac.21660 33538338

[2] Wolfgang CL, Herman JM, Laheru DA, Klein AP, Erdek MA, Fishman EK, Hruban RH. Recent progress in pancreatic cancer. CA Cancer J Clin. 2013; 63:318–48. 10.3322/caac.21190 23856911 PMC3769458

[3] Goral V. Pancreatic Cancer: Pathogenesis and Diagnosis. Asian Pac J Cancer Prev. 2015; 16:5619–24. 10.7314/apjcp.2015.16.14.5619 26320426

[4] Vincent A, Herman J, Schulick R, Hruban RH, Goggins M. Pancreatic cancer. Lancet. 2011; 378:607–20. 10.1016/S0140-6736(10)62307-0 21620466 PMC3062508

[5] Singhi AD, Koay EJ, Chari ST, Maitra A. Early Detection of Pancreatic Cancer: Opportunities and Challenges. Gastroenterology. 2019; 156:2024–40. 10.1053/j.gastro.2019.01.259 30721664 PMC6486851

[6] Hidalgo M, Cascinu S, Kleeff J, Labianca R, Löhr JM, Neoptolemos J, Real FX, Van Laethem JL, Heinemann V. Addressing the challenges of pancreatic cancer: future directions for improving outcomes. Pancreatology. 2015; 15:8–18. 10.1016/j.pan.2014.10.001 25547205

[7] Bear AS, Vonderheide RH, O'Hara MH. Challenges and Opportunities for Pancreatic Cancer Immunotherapy. Cancer Cell. 2020; 38:788–802. 10.1016/j.ccell.2020.08.004 32946773 PMC7738380

[8] O'Reilly EM, Oh DY, Dhani N, Renouf DJ, Lee MA, Sun W, Fisher G, Hezel A, Chang SC, Vlahovic G, Takahashi O, Yang Y, Fitts D, Philip PA. Durvalumab With or Without Tremelimumab for Patients With Metastatic Pancreatic Ductal Adenocarcinoma: A Phase 2 Randomized Clinical Trial. JAMA Oncol. 2019; 5:1431–8. 10.1001/jamaoncol.2019.1588 31318392 PMC6647002

[9] Zhou Y, Jin X, Yu H, Qin G, Pan P, Zhao J, Chen T, Liang X, Sun Y, Wang B, Ren D, Zhu S, Wu H. HDAC5 modulates PD-L1 expression and cancer immunity via p65 deacetylation in pancreatic cancer. Theranostics. 2022; 12:2080–94. 10.7150/thno.69444 35265200 PMC8899586

[10] Zhang K, Corsa CA, Ponik SM, Prior JL, Piwnica-Worms D, Eliceiri KW, Keely PJ, Longmore GD. The collagen receptor discoidin domain receptor 2 stabilizes SNAIL1 to facilitate breast cancer metastasis. Nat Cell Biol. 2013; 15:677–87. 10.1038/ncb2743 23644467 PMC3794710

[11] Ren B, Cui M, Yang G, Wang H, Feng M, You L, Zhao Y. Tumor microenvironment participates in metastasis of pancreatic cancer. Mol Cancer. 2018; 17:108. 10.1186/s12943-018-0858-1 30060755 PMC6065152

[12] Zhang X, Shi M, Chen T, Zhang B. Characterization of the Immune Cell Infiltration Landscape in Head and Neck Squamous Cell Carcinoma to Aid Immunotherapy. Mol Ther Nucleic Acids. 2020; 22:298–309. 10.1016/j.omtn.2020.08.030 33230435 PMC7522342

[13] Chen S, Huang F, Chen S, Chen Y, Li J, Li Y, Lian G, Huang K. Bioinformatics-Based Identification of Tumor Microenvironment-Related Prognostic Genes in Pancreatic Cancer. Front Genet. 2021; 12:632803. 10.3389/fgene.2021.632803 34276760 PMC8277941

[14] Shen L, Li J, Liu Q, Song W, Zhang X, Tiruthani K, Hu H, Das M, Goodwin TJ, Liu R, Huang L. Local Blockade of Interleukin 10 and C-X-C Motif Chemokine Ligand 12 with Nano-Delivery Promotes Antitumor Response in Murine Cancers. ACS Nano. 2018; 12:9830–41. 10.1021/acsnano.8b00967 30253648

[15] Pagès F, Mlecnik B, Marliot F, Bindea G, Ou FS, Bifulco C, Lugli A, Zlobec I, Rau TT, Berger MD, Nagtegaal ID, Vink-Börger E, Hartmann A, et al. International validation of the consensus Immunoscore for the classification of colon cancer: a prognostic and accuracy study. Lancet. 2018; 391:2128–39. 10.1016/S0140-6736(18)30789-X 29754777

[16] Galdiero MR, Bianchi P, Grizzi F, Di Caro G, Basso G, Ponzetta A, Bonavita E, Barbagallo M, Tartari S, Polentarutti N, Malesci A, Marone G, Roncalli M, et al. Occurrence and significance of tumor-associated neutrophils in patients with colorectal cancer. Int J Cancer. 2016; 139:446–56. 10.1002/ijc.30076 26939802

[17] Jensen TO, Schmidt H, Møller HJ, Donskov F, Høyer M, Sjoegren P, Christensen IJ, Steiniche T. Intratumoral neutrophils and plasmacytoid dendritic cells indicate poor prognosis and are associated with pSTAT3 expression in AJCC stage I/II melanoma. Cancer. 2012; 118:2476–85. 10.1002/cncr.26511 21953023

[18] Yuen KC, Liu LF, Gupta V, Madireddi S, Keerthivasan S, Li C, Rishipathak D, Williams P, Kadel EE 3rd, Koeppen H, Chen YJ, Modrusan Z, Grogan JL, et al. High systemic and tumor-associated IL-8 correlates with reduced clinical benefit of PD-L1 blockade. Nat Med. 2020; 26:693–8. 10.1038/s41591-020-0860-1 32405063 PMC8286544

[19] Steele CW, Karim SA, Leach JDG, Bailey P, Upstill-Goddard R, Rishi L, Foth M, Bryson S, McDaid K, Wilson Z, Eberlein C, Candido JB, Clarke M, et al. CXCR2 Inhibition Profoundly Suppresses Metastases and Augments Immunotherapy in Pancreatic Ductal Adenocarcinoma. Cancer Cell. 2016; 29:832–45. 10.1016/j.ccell.2016.04.014 27265504 PMC4912354

[20] Nywening TM, Belt BA, Cullinan DR, Panni RZ, Han BJ, Sanford DE, Jacobs RC, Ye J, Patel AA, Gillanders WE, Fields RC, DeNardo DG, Hawkins WG, et al. Targeting both tumour-associated CXCR2^+^ neutrophils and CCR2^+^ macrophages disrupts myeloid recruitment and improves chemotherapeutic responses in pancreatic ductal adenocarcinoma. Gut. 2018; 67:1112–23. 10.1136/gutjnl-2017-313738 29196437 PMC5969359

[21] Hosoi F, Izumi H, Kawahara A, Murakami Y, Kinoshita H, Kage M, Nishio K, Kohno K, Kuwano M, Ono M. N-myc downstream regulated gene 1/Cap43 suppresses tumor growth and angiogenesis of pancreatic cancer through attenuation of inhibitor of kappaB kinase beta expression. Cancer Res. 2009; 69:4983–91. 10.1158/0008-5472.CAN-08-4882 19491262

[22] Zhang Y, Chandra V, Riquelme Sanchez E, Dutta P, Quesada PR, Rakoski A, Zoltan M, Arora N, Baydogan S, Horne W, Burks J, Xu H, Hussain P, et al. Interleukin-17-induced neutrophil extracellular traps mediate resistance to checkpoint blockade in pancreatic cancer. J Exp Med. 2020; 217:e20190354. 10.1084/jem.20190354 32860704 PMC7953739

[23] Fridlender ZG, Sun J, Kim S, Kapoor V, Cheng G, Ling L, Worthen GS, Albelda SM. Polarization of tumor-associated neutrophil phenotype by TGF-beta: "N1" versus "N2" TAN. Cancer Cell. 2009; 16:183–94. 10.1016/j.ccr.2009.06.017 19732719 PMC2754404

[24] Jaillon S, Ponzetta A, Di Mitri D, Santoni A, Bonecchi R, Mantovani A. Neutrophil diversity and plasticity in tumour progression and therapy. Nat Rev Cancer. 2020; 20:485–503. 10.1038/s41568-020-0281-y 32694624

[25] Wang X, Qiu L, Li Z, Wang XY, Yi H. Understanding the Multifaceted Role of Neutrophils in Cancer and Autoimmune Diseases. Front Immunol. 2018; 9:2456. 10.3389/fimmu.2018.02456 30473691 PMC6237929

[26] Shojaei F, Wu X, Zhong C, Yu L, Liang XH, Yao J, Blanchard D, Bais C, Peale FV, van Bruggen N, Ho C, Ross J, Tan M, et al. Bv8 regulates myeloid-cell-dependent tumour angiogenesis. Nature. 2007; 450:825–31. 10.1038/nature06348 18064003

[27] Schmielau J, Finn OJ. Activated granulocytes and granulocyte-derived hydrogen peroxide are the underlying mechanism of suppression of t-cell function in advanced cancer patients. Cancer Res. 2001; 61:4756–60. 11406548

[28] Jin L, Kim HS, Shi J. Neutrophil in the Pancreatic Tumor Microenvironment. Biomolecules. 2021; 11:1170. 10.3390/biom11081170 34439836 PMC8394314

[29] Stromnes IM, Brockenbrough JS, Izeradjene K, Carlson MA, Cuevas C, Simmons RM, Greenberg PD, Hingorani SR. Targeted depletion of an MDSC subset unmasks pancreatic ductal adenocarcinoma to adaptive immunity. Gut. 2014; 63:1769–81. 10.1136/gutjnl-2013-306271 24555999 PMC4340484

[30] Li C, Cui L, Yang L, Wang B, Zhuo Y, Zhang L, Wang X, Zhang Q, Zhang S. Pancreatic Stellate Cells Promote Tumor Progression by Promoting an Immunosuppressive Microenvironment in Murine Models of Pancreatic Cancer. Pancreas. 2020; 49:120–7. 10.1097/MPA.0000000000001464 31856087

[31] Hu H, Hang JJ, Han T, Zhuo M, Jiao F, Wang LW. The M2 phenotype of tumor-associated macrophages in the stroma confers a poor prognosis in pancreatic cancer. Tumour Biol. 2016; 37:8657–64. 10.1007/s13277-015-4741-z 26738860

[32] Moncada R, Barkley D, Wagner F, Chiodin M, Devlin JC, Baron M, Hajdu CH, Simeone DM, Yanai I. Integrating microarray-based spatial transcriptomics and single-cell RNA-seq reveals tissue architecture in pancreatic ductal adenocarcinomas. Nat Biotechnol. 2020; 38:333–42. 10.1038/s41587-019-0392-8 31932730

[33] Tomczak K, Czerwińska P, Wiznerowicz M. The Cancer Genome Atlas (TCGA): an immeasurable source of knowledge. Contemp Oncol (Pozn). 2015; 19:A68–77. 10.5114/wo.2014.47136 25691825 PMC4322527

[34] Zhang G, Schetter A, He P, Funamizu N, Gaedcke J, Ghadimi BM, Ried T, Hassan R, Yfantis HG, Lee DH, Lacy C, Maitra A, Hanna N, et al. DPEP1 inhibits tumor cell invasiveness, enhances chemosensitivity and predicts clinical outcome in pancreatic ductal adenocarcinoma. PLoS One. 2012; 7:e31507. 10.1371/journal.pone.0031507 22363658 PMC3282755

[35] Zhang G, He P, Tan H, Budhu A, Gaedcke J, Ghadimi BM, Ried T, Yfantis HG, Lee DH, Maitra A, Hanna N, Alexander HR, Hussain SP. Integration of metabolomics and transcriptomics revealed a fatty acid network exerting growth inhibitory effects in human pancreatic cancer. Clin Cancer Res. 2013; 19:4983–93. 10.1158/1078-0432.CCR-13-0209 23918603 PMC3778077

[36] Yang S, He P, Wang J, Schetter A, Tang W, Funamizu N, Yanaga K, Uwagawa T, Satoskar AR, Gaedcke J, Bernhardt M, Ghadimi BM, Gaida MM, et al. A Novel MIF Signaling Pathway Drives the Malignant Character of Pancreatic Cancer by Targeting NR3C2. Cancer Res. 2016; 76:3838–50. 10.1158/0008-5472.CAN-15-2841 27197190 PMC4930741

[37] Chen DT, Davis-Yadley AH, Huang PY, Husain K, Centeno BA, Permuth-Wey J, Pimiento JM, Malafa M. Prognostic Fifteen-Gene Signature for Early Stage Pancreatic Ductal Adenocarcinoma. PLoS One. 2015; 10:e0133562. 10.1371/journal.pone.0133562 26247463 PMC4527782

[38] Leek JT, Johnson WE, Parker HS, Jaffe AE, Storey JD. The sva package for removing batch effects and other unwanted variation in high-throughput experiments. Bioinformatics. 2012; 28:882–3. 10.1093/bioinformatics/bts034 22257669 PMC3307112

[39] Yuan Q, Zhang W, Shang W. Identification and validation of a prognostic risk-scoring model based on sphingolipid metabolism-associated cluster in colon adenocarcinoma. Front Endocrinol (Lausanne). 2022; 13:1045167. 10.3389/fendo.2022.1045167 36518255 PMC9742378

[40] Butler A, Hoffman P, Smibert P, Papalexi E, Satija R. Integrating single-cell transcriptomic data across different conditions, technologies, and species. Nat Biotechnol. 2018; 36:411–20. 10.1038/nbt.4096 29608179 PMC6700744

[41] Liu J, Yuan Q, Ren J, Li Y, Zhang Y, Shang D. Single-cell sequencing and bulk RNA sequencing reveal a cell differentiation-related multigene panel to predict the prognosis and immunotherapy response of hepatocellular carcinoma. Chin Med J (Engl). 2023; 136:485–7. 10.1097/CM9.0000000000002393 36723861 PMC10106201

[42] Mabbott NA, Baillie JK, Brown H, Freeman TC, Hume DA. An expression atlas of human primary cells: inference of gene function from coexpression networks. BMC Genomics. 2013; 14:632. 10.1186/1471-2164-14-632 24053356 PMC3849585

[43] Yu G, Wang LG, Han Y, He QY. clusterProfiler: an R package for comparing biological themes among gene clusters. OMICS. 2012; 16:284–7. 10.1089/omi.2011.0118 22455463 PMC3339379

[44] Wilkerson MD, Hayes DN. ConsensusClusterPlus: a class discovery tool with confidence assessments and item tracking. Bioinformatics. 2010; 26:1572–3. 10.1093/bioinformatics/btq170 20427518 PMC2881355

[45] Zhang B, Yuan Q, Zhang B, Li S, Wang Z, Liu H, Meng F, Chen X, Shang D. Characterization of neuroendocrine regulation- and metabolism-associated molecular features and prognostic indicators with aid to clinical chemotherapy and immunotherapy of patients with pancreatic cancer. Front Endocrinol (Lausanne). 2023; 13:1078424. 10.3389/fendo.2022.1078424 36743929 PMC9895410

[46] Chi H, Yang J, Peng G, Zhang J, Song G, Xie X, Xia Z, Liu J, Tian G. Circadian rhythm-related genes index: A predictor for HNSCC prognosis, immunotherapy efficacy, and chemosensitivity. Front Immunol. 2023; 14:1091218. 10.3389/fimmu.2023.1091218 36969232 PMC10036372

[47] Liu S, Wang Z, Zhu R, Wang F, Cheng Y, Liu Y. Three Differential Expression Analysis Methods for RNA Sequencing: limma, EdgeR, DESeq2. J Vis Exp. 2021. 10.3791/62528 34605806

[48] Chi H, Zhao S, Yang J, Gao X, Peng G, Zhang J, Xie X, Song G, Xu K, Xia Z, Chen S, Zhao J. T-cell exhaustion signatures characterize the immune landscape and predict HCC prognosis via integrating single-cell RNA-seq and bulk RNA-sequencing. Front Immunol. 2023; 14:1137025. 10.3389/fimmu.2023.1137025 37006257 PMC10050519

[49] Wang J, Ren J, Liu J, Zhang L, Yuan Q, Dong B. Identification and verification of the ferroptosis- and pyroptosis-associated prognostic signature for low-grade glioma. Bosn J Basic Med Sci. 2022; 22:728–50. 10.17305/bjbms.2021.6888 35276059 PMC9519161

[50] Zhao S, Chi H, Yang Q, Chen S, Wu C, Lai G, Xu K, Su K, Luo H, Peng G, Xia Z, Cheng C, Lu P. Identification and validation of neurotrophic factor-related gene signatures in glioblastoma and Parkinson's disease. Front Immunol. 2023; 14:1090040. 10.3389/fimmu.2023.1090040 36825022 PMC9941742

[51] Zhao S, Zhang X, Gao F, Chi H, Zhang J, Xia Z, Cheng C, Liu J. Identification of copper metabolism-related subtypes and establishment of the prognostic model in ovarian cancer. Front Endocrinol (Lausanne). 2023; 14:1145797. 10.3389/fendo.2023.1145797 36950684 PMC10025496

[52] Subramanian A, Tamayo P, Mootha VK, Mukherjee S, Ebert BL, Gillette MA, Paulovich A, Pomeroy SL, Golub TR, Lander ES, Mesirov JP. Gene set enrichment analysis: a knowledge-based approach for interpreting genome-wide expression profiles. Proc Natl Acad Sci U S A. 2005; 102:15545–50. 10.1073/pnas.0506580102 16199517 PMC1239896

[53] Hänzelmann S, Castelo R, Guinney J. GSVA: gene set variation analysis for microarray and RNA-seq data. BMC Bioinformatics. 2013; 14:7. 10.1186/1471-2105-14-7 23323831 PMC3618321

[54] Zhang L, Zhao Y, Dai Y, Cheng JN, Gong Z, Feng Y, Sun C, Jia Q, Zhu B. Immune Landscape of Colorectal Cancer Tumor Microenvironment from Different Primary Tumor Location. Front Immunol. 2018; 9:1578. 10.3389/fimmu.2018.01578 30042763 PMC6048410

[55] Newman AM, Liu CL, Green MR, Gentles AJ, Feng W, Xu Y, Hoang CD, Diehn M, Alizadeh AA. Robust enumeration of cell subsets from tissue expression profiles. Nat Methods. 2015; 12:453–7. 10.1038/nmeth.3337 25822800 PMC4739640

[56] Maeser D, Gruener RF, Huang RS. oncoPredict: an R package for predicting in vivo or cancer patient drug response and biomarkers from cell line screening data. Brief Bioinform. 2021; 22:bbab260. 10.1093/bib/bbab260 34260682 PMC8574972

[57] Tang Z, Li C, Kang B, Gao G, Li C, Zhang Z. GEPIA: a web server for cancer and normal gene expression profiling and interactive analyses. Nucleic Acids Res. 2017; 45:W98–102. 10.1093/nar/gkx247 28407145 PMC5570223

[58] Uhlen M, Zhang C, Lee S, Sjöstedt E, Fagerberg L, Bidkhori G, Benfeitas R, Arif M, Liu Z, Edfors F, Sanli K, von Feilitzen K, Oksvold P, et al. A pathology atlas of the human cancer transcriptome. Science. 2017; 357:eaan2507. 10.1126/science.aan2507 28818916

[59] Chandrashekar DS, Karthikeyan SK, Korla PK, Patel H, Shovon AR, Athar M, Netto GJ, Qin ZS, Kumar S, Manne U, Creighton CJ, Varambally S. UALCAN: An update to the integrated cancer data analysis platform. Neoplasia. 2022; 25:18–27. 10.1016/j.neo.2022.01.001 35078134 PMC8788199

[60] Chandrashekar DS, Bashel B, Balasubramanya SAH, Creighton CJ, Ponce-Rodriguez I, Chakravarthi BVS, Varambally S. UALCAN: A Portal for Facilitating Tumor Subgroup Gene Expression and Survival Analyses. Neoplasia. 2017; 19:649–58. 10.1016/j.neo.2017.05.002 28732212 PMC5516091

[61] Delitto D, Wallet SM, Hughes SJ. Targeting tumor tolerance: A new hope for pancreatic cancer therapy? Pharmacol Ther. 2016; 166:9–29. 10.1016/j.pharmthera.2016.06.008 27343757

[62] Mace TA, Ameen Z, Collins A, Wojcik S, Mair M, Young GS, Fuchs JR, Eubank TD, Frankel WL, Bekaii-Saab T, Bloomston M, Lesinski GB. Pancreatic cancer-associated stellate cells promote differentiation of myeloid-derived suppressor cells in a STAT3-dependent manner. Cancer Res. 2013; 73:3007–18. 10.1158/0008-5472.CAN-12-4601 23514705 PMC3785672

[63] Cai J, Chen H, Lu M, Zhang Y, Lu B, You L, Zhang T, Dai M, Zhao Y. Advances in the epidemiology of pancreatic cancer: Trends, risk factors, screening, and prognosis. Cancer Lett. 2021; 520:1–11. 10.1016/j.canlet.2021.06.027 34216688

[64] Jin W, Yin H, Li H, Yu XJ, Xu HX, Liu L. Neutrophil extracellular DNA traps promote pancreatic cancer cells migration and invasion by activating EGFR/ERK pathway. J Cell Mol Med. 2021; 25:5443–56. 10.1111/jcmm.16555 33955688 PMC8184670

[65] Jung HS, Gu J, Kim JE, Nam Y, Song JW, Kim HK. Cancer cell-induced neutrophil extracellular traps promote both hypercoagulability and cancer progression. PLoS One. 2019; 14:e0216055. 10.1371/journal.pone.0216055 31034495 PMC6488070

[66] Abdol Razak N, Elaskalani O, Metharom P. Pancreatic Cancer-Induced Neutrophil Extracellular Traps: A Potential Contributor to Cancer-Associated Thrombosis. Int J Mol Sci. 2017; 18:487. 10.3390/ijms18030487 28245569 PMC5372503

[67] Albrengues J, Shields MA, Ng D, Park CG, Ambrico A, Poindexter ME, Upadhyay P, Uyeminami DL, Pommier A, Küttner V, Bružas E, Maiorino L, Bautista C, et al. Neutrophil extracellular traps produced during inflammation awaken dormant cancer cells in mice. Science. 2018; 361:eaao4227. 10.1126/science.aao4227 30262472 PMC6777850

[68] Ren X, Kang B, Zhang Z. Understanding tumor ecosystems by single-cell sequencing: promises and limitations. Genome Biol. 2018; 19:211. 10.1186/s13059-018-1593-z 30509292 PMC6276232

[69] Liang J, Chen Z, Huang Y, Bi G, Bian Y, Jin X, Zhang H, Sui Q, Zhan C, Wang Q. Signatures of malignant cells and novel therapeutic targets revealed by single-cell sequencing in lung adenocarcinoma. Cancer Med. 2022; 11:2244–58. 10.1002/cam4.4547 35102706 PMC9160812

[70] Newman AM, Steen CB, Liu CL, Gentles AJ, Chaudhuri AA, Scherer F, Khodadoust MS, Esfahani MS, Luca BA, Steiner D, Diehn M, Alizadeh AA. Determining cell type abundance and expression from bulk tissues with digital cytometry. Nat Biotechnol. 2019; 37:773–82. 10.1038/s41587-019-0114-2 31061481 PMC6610714

[71] Tas F, Aykan F, Alici S, Kaytan E, Aydiner A, Topuz E. Prognostic factors in pancreatic carcinoma: serum LDH levels predict survival in metastatic disease. Am J Clin Oncol. 2001; 24:547–50. 10.1097/00000421-200112000-00003 11801751

[72] Stocken DD, Hassan AB, Altman DG, Billingham LJ, Bramhall SR, Johnson PJ, Freemantle N. Modelling prognostic factors in advanced pancreatic cancer. Br J Cancer. 2008; 99:883–93. 10.1038/sj.bjc.6604568 19238630 PMC2538756

[73] Cheng CS, Tan HY, Wang N, Chen L, Meng Z, Chen Z, Feng Y. Functional inhibition of lactate dehydrogenase suppresses pancreatic adenocarcinoma progression. Clin Transl Med. 2021; 11:e467. 10.1002/ctm2.467 34185423 PMC8238920

[74] Maftouh M, Avan A, Sciarrillo R, Granchi C, Leon LG, Rani R, Funel N, Smid K, Honeywell R, Boggi U, Minutolo F, Peters GJ, Giovannetti E. Synergistic interaction of novel lactate dehydrogenase inhibitors with gemcitabine against pancreatic cancer cells in hypoxia. Br J Cancer. 2014; 110:172–82. 10.1038/bjc.2013.681 24178759 PMC3887288

[75] Yu SL, Xu LT, Qi Q, Geng YW, Chen H, Meng ZQ, Wang P, Chen Z. Serum lactate dehydrogenase predicts prognosis and correlates with systemic inflammatory response in patients with advanced pancreatic cancer after gemcitabine-based chemotherapy. Sci Rep. 2017; 7:45194. 10.1038/srep45194 28345594 PMC5366928

[76] Li J, Zhu S, Tong J, Hao H, Yang J, Liu Z, Wang Y. Suppression of lactate dehydrogenase A compromises tumor progression by downregulation of the Warburg effect in glioblastoma. Neuroreport. 2016; 27:110–5. 10.1097/WNR.0000000000000506 26694942 PMC4712768

[77] Zhao J, Huang X, Xu Z, Dai J, He H, Zhu Y, Wang H. LDHA promotes tumor metastasis by facilitating epithelial-mesenchymal transition in renal cell carcinoma. Mol Med Rep. 2017; 16:8335–44. 10.3892/mmr.2017.7637 28983605

[78] Mantovani A, Barajon I, Garlanda C. IL-1 and IL-1 regulatory pathways in cancer progression and therapy. Immunol Rev. 2018; 281:57–61. 10.1111/imr.12614 29247996 PMC5922413

[79] Liu Y, Xing Z, Yuan M, Xu B, Chen L, Zhang D, Zhou Y, Huang H, Zheng X, Zhang J, Jiang J. IL1R2 promotes tumor progression via JAK2/STAT3 pathway in human clear cell renal cell carcinoma. Pathol Res Pract. 2022; 238:154069. 10.1016/j.prp.2022.154069 36029680

[80] Mar AC, Chu CH, Lee HJ, Chien CW, Cheng JJ, Yang SH, Jiang JK, Lee TC. Interleukin-1 Receptor Type 2 Acts with c-Fos to Enhance the Expression of Interleukin-6 and Vascular Endothelial Growth Factor A in Colon Cancer Cells and Induce Angiogenesis. J Biol Chem. 2015; 290:22212–24. 10.1074/jbc.M115.644823 26209639 PMC4571972

[81] Yamaki M, Sugiura K, Muro Y, Shimoyama Y, Tomita Y. Epidermal growth factor receptor tyrosine kinase inhibitors induce CCL2 and CCL5 via reduction in IL-1R2 in keratinocytes. Exp Dermatol. 2010; 19:730–5. 10.1111/j.1600-0625.2010.01108.x 20590818

[82] Shimizu K, Nakajima A, Sudo K, Liu Y, Mizoroki A, Ikarashi T, Horai R, Kakuta S, Watanabe T, Iwakura Y. IL-1 receptor type 2 suppresses collagen-induced arthritis by inhibiting IL-1 signal on macrophages. J Immunol. 2015; 194:3156–68. 10.4049/jimmunol.1402155 25725107

[83] Liu X, Min L, Duan H, Shi R, Zhang W, Hong S, Tu C. Short hairpin RNA (shRNA) of type 2 interleukin-1 receptor (IL1R2) inhibits the proliferation of human osteosarcoma U-2 OS cells. Med Oncol. 2015; 32:364. 10.1007/s12032-014-0364-2 25432697

[84] Oelmann E, Stein H, Berdel WE, Herbst H. Expression of Interleukin-1 and Interleukin-1 Receptors Type 1 and Type 2 in Hodgkin Lymphoma. PLoS One. 2015; 10:e0138747. 10.1371/journal.pone.0138747 26406983 PMC4583993

[85] Tang Q, Chen J, Di Z, Yuan W, Zhou Z, Liu Z, Han S, Liu Y, Ying G, Shu X, Di M. TM4SF1 promotes EMT and cancer stemness via the Wnt/β-catenin/SOX2 pathway in colorectal cancer. J Exp Clin Cancer Res. 2020; 39:232. 10.1186/s13046-020-01690-z 33153498 PMC7643364

[86] Cao J, Yang J, Ramachandran V, Arumugam T, Deng D, Li Z, Xu L, Logsdon CD. TM4SF1 Promotes Gemcitabine Resistance of Pancreatic Cancer In Vitro and In Vivo. PLoS One. 2015; 10:e0144969. 10.1371/journal.pone.0144969 26709920 PMC4692438

[87] Huang YK, Fan XG, Qiu F. TM4SF1 Promotes Proliferation, Invasion, and Metastasis in Human Liver Cancer Cells. Int J Mol Sci. 2016; 17:661. 10.3390/ijms17050661 27153056 PMC4881487

[88] Zheng YW, Wang M, Zhong ZM, Wu GQ, Zhang T, Chen LL, Li M. TM4SF1 promotes glioma malignancy through multiple mechanisms. Neoplasma. 2022; 69:859–67. 10.4149/neo_2022_211009N1427 35532297

[89] Pan T, Sun S, Chen Y, Tian R, Chen E, Tan R, Wang X, Liu Z, Liu J, Qu H. Immune effects of PI3K/Akt/HIF-1α-regulated glycolysis in polymorphonuclear neutrophils during sepsis. Crit Care. 2022; 26:29. 10.1186/s13054-022-03893-6 35090526 PMC8796568

[90] Herremans KM, Szymkiewicz DD, Riner AN, Bohan RP, Tushoski GW, Davidson AM, Lou X, Leong MC, Dean BD, Gerber M, Underwood PW, Han S, Hughes SJ. The interleukin-1 axis and the tumor immune microenvironment in pancreatic ductal adenocarcinoma. Neoplasia. 2022; 28:100789. 10.1016/j.neo.2022.100789 35395492 PMC8990176

[91] Wright MD, Moseley GW, van Spriel AB. Tetraspanin microdomains in immune cell signalling and malignant disease. Tissue Antigens. 2004; 64:533–42. 10.1111/j.1399-0039.2004.00321.x 15496196

