# Supplementary material for: Integrated single-cell and bulk RNA sequencing revealed the molecular characteristics and prognostic roles of neutrophils in pancreatic cancer
Source: Aging (Albany NY). 2023 Sep 19;15(18):9718–42. doi: 10.18632/aging.205044 (PMC10564426; doi:10.18632/aging.205044)
Supplement: Supplementary Figure 1 [file aging-15-205044-s001.pdf]

SUPPLEMENTARY FIGURE

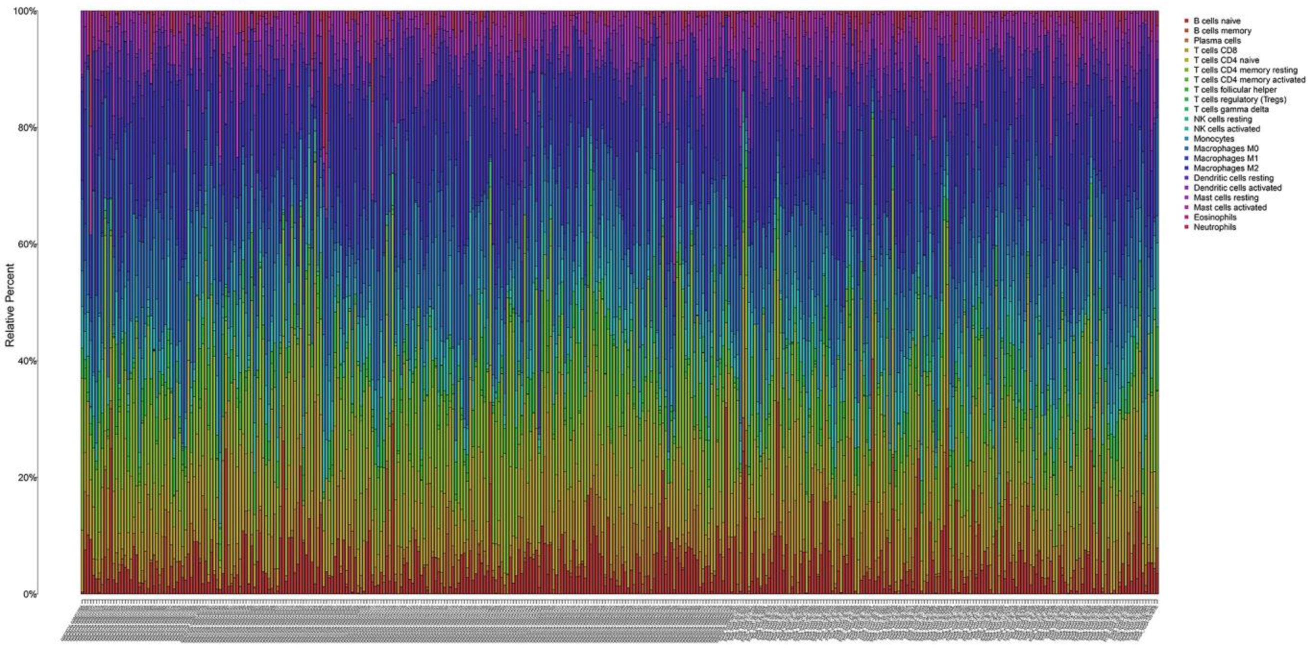

Supplementary Figure 1. Stacked bar plots of the distribution of 22 immune cell subspecies in each sample.
